# Biogeography of Anurans from the Poorly Known and Threatened Coastal Sandplains of Eastern Brazil

**DOI:** 10.1371/journal.pone.0128268

**Published:** 2015-06-05

**Authors:** Ariane Lima Xavier, Thaís Barreto Guedes, Marcelo Felgueiras Napoli

**Affiliations:** 1 Programa de Pós-Graduação em Ecologia e Biomonitoramento, Instituto de Biologia, Universidade Federal da Bahia, Salvador, Bahia, Brasil; 2 Instituto Federal de Educação, Ciência e Tecnologia Baiano, Valença, Bahia, Brasil; 3 Programa de Pós-Graduação em Ecologia e Evolução, Universidade Federal de São Paulo, Diadema, São Paulo, Brasil; 4 Museu de Zoologia, Universidade de São Paulo, São Paulo, São Paulo, Brasil; 5 Museu de Zoologia, Departamento de Zoologia, Instituto de Biologia, Universidade Federal da Bahia, Salvador, Bahia, Brasil; 6 Research associate at Departamento de Vertebrados, Museu Nacional, Universidade Federal do Rio de Janeiro, Rio de Janeiro, Rio de Janeiro, Brasil; Trier University, GERMANY

## Abstract

The east coast of Brazil comprises an extensive area inserted in the Tropical Atlantic Domain and is represented by sandy plains of beach ridges commonly known as Restingas. The coastal environments are unique and house a rich amphibian fauna, the geographical distribution patterns of which are incipient. Biogeographical studies can explain the current distributional patterns and provide the identification of natural biogeographical units. These areas are important in elucidating the evolutionary history of the taxa and the areas where they occur. The aim of this study was to seek natural biogeographical units in the Brazilian sandy plains of beach ridges by means of distribution data of amphibians and to test the main predictions of the vicariance model to explain the patterns found. We revised and georeferenced data on the geographical distribution of 63 anuran species. We performed a search for latitudinal distribution patterns along the sandy coastal plains of Brazil using the non-metric multidimensional scaling method (NMDS) and the biotic element analysis to identify natural biogeographical units. The results showed a monotonic variation in anuran species composition along the latitudinal gradient with a break in the clinal pattern from 23°S to 25°S latitude (states of Rio de Janeiro to São Paulo). The major predictions of the vicariance model were corroborated by the detection of four biotic elements with significantly clustered distribution and by the presence of congeneric species distributed in distinct biotic elements. The results support the hypothesis that vicariance could be one of the factors responsible for the distribution patterns of the anuran communities along the sandy coastal plains of eastern Brazil. The results of the clusters are also congruent with the predictions of paleoclimatic models made for the Last Glacial Maximum of the Pleistocene, such as the presence of historical forest refugia and biogeographical patterns already detected for amphibians in the Atlantic Rainforest.

## Introduction

Biogeography is the discipline interested in documenting and understanding spatial biodiversity patterns [1] and also in explaining the evolutionary history that led to this current spatial configuration [2–5]. Detailed data regarding how organisms are distributed, the basis of biogeographical studies, enable such distribution patterns to be identified, including natural biogeographical units [6–11]. These natural biogeographical regions are fundamental units of comparison in many broad-scale ecological and evolutionary studies [12,13] and provide an essential tool for conservation planning [11,14–18].

There are several methods proposed to identify biogeographical units (e.g., [6,19–22]). A well-known method is the parsimony analysis of endemicity that is used to detect natural biogeographical units in named areas of endemism [6,9,23,24]. According to some authors (e.g., [1,5,25]), areas of endemism have a unique biota with similar historical processes and are the basis for postulating hypotheses regarding the processes that led to their origin. However, the determination of natural biogeographical units based solely on strict endemism is effective only in cases of strict sympatry [19,21] that is not so common in natural conditions. Dispersal and extinction are natural events that can cause noise in the identification of areas of endemism and hinder the recognition of natural biogeographical units [20,22]. For this reason, biotic element analysis has been used in many studies as an alternative method to detect natural biogeographical units (e.g., [10,11,19,21,26–28]).

The biotic element analysis identifies groups of taxa with geographic distributions significantly more similar to one another [19,21]. The advantage is that biotic elements may be recognized even when part of the taxa originated by vicariance has dispersed across barriers [19,21]. The biotic element analysis is based on the assumption of vicariance and postulates that diversification results from fragmentation of the ancestral biota by the emergence of barriers [5,19,21,29,30]. Consequently, it is expected that the distributions of taxa with the same geographical origin are more similar to each other than to the distributions of taxa from distinct geographical origins, and the taxa that are closely related due to the vicariance process belong to distinct biotic elements [19,21].

The identification of natural biogeographical units is important to understand the evolutionary history of taxa and of the areas that encompass them, and such studies in natural environments are incipient [11,28], as in the case of the biota from the sandy plains of the coastal ridges of Brazil. The coastal sandplains are commonly known as Restingas and are included in the Tropical Atlantic Domain [31], which also includes the Atlantic Forest, a global biodiversity hotspot [32]. Studies on different biological groups, especially those focused on forest habitats of the Atlantic Forest in Brazil, have been carried out to identify distributional patterns [7,33,34].

Biogeographic studies have not yet addressed the distribution patterns of amphibian communities or the processes that have shaped these communities. Additionally, studies in the Restingas area of the Tropical Atlantic Domain is neglected, as most investigations have focused on the forested part of this domain [35–39]. For this reason, we assessed for the first time the amphibian distribution patterns in a biogeographical study of Restingas. The aims of our study were: (1) to identify distribution patterns of anuran species occurring on sandy plains of beach ridges of the eastern Brazilian coast; (2) to detect natural biogeographical units throughout the study area and to identify groups of anuran species with non-random overlapping geographical distribution (biotic elements); and (3) to provide the first formal test of two predictions of the vicariance model to evaluate the hypothesis that the diversification of these species can be a result of fragmentation of the ancestral biota by emerging barriers.

## Materials and Methods

### Study area

**Fig 1:**
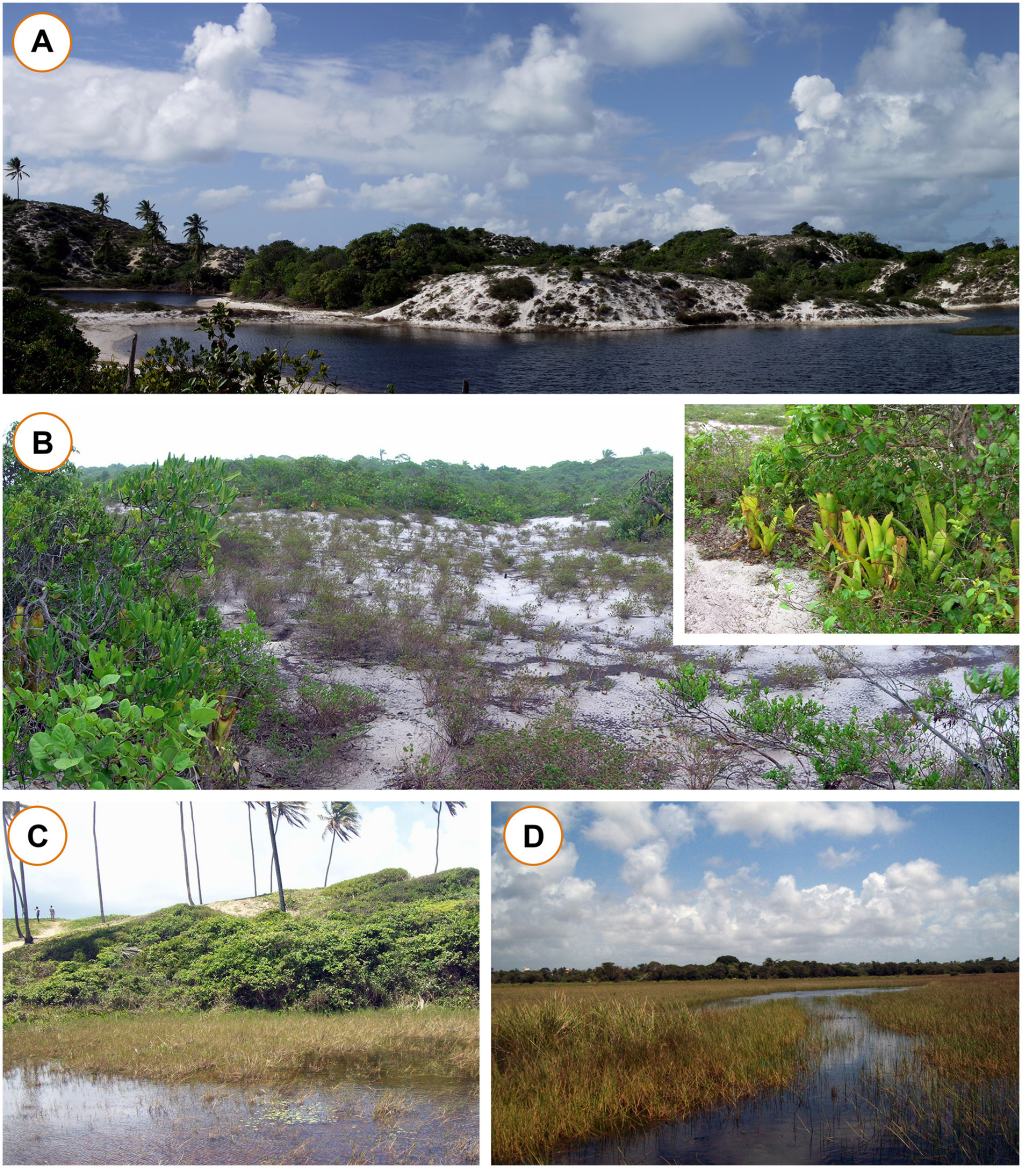
Phytophysiognomies of the Restinga. (A) Panoramic view of the Restinga from Costa Azul, Municipality of Jandaíra, northernmost coast of the Bahia State, northeastern Brazil, featuring freshwater ponds amid sand dunes. (B) A typical Restinga landscape (Praia do Forte, Municipality of Mata de São João, Bahia State) characterized by sandy soil covered by many shrubs and terrestrial bromeliads. In several places of the Restinga habitat, terrestrial bromeliads (in detail in the top right corner) are the unique source of water and shelter for anuran species. (C) Temporary pond near a sand dune and (D) floodplain in the Restinga of Arembepe, Municipality of Camaçari, Bahia State. These floodplains are contiguous with sandplains and many amphibian species inhabiting the Restingas commonly use these areas for reproduction and foraging. Photo A, Ariane L. Xavier, 10 June 2012; B, Rafael O. Abreu, 17 June 2006; C and D, Iris Shalon F. Carneiro, 28 September 2014.

The east coast of Brazil is located within the Tropical Atlantic Domain [31] and is essentially covered by the Atlantic Forest, a global biodiversity hotspot [32]. The Restinga is a component of the Atlantic Forest habitat characterized by dunes and sandy plains covered by herbaceous and shrubby vegetation under direct sunlight (‘open Restinga’, Fig 1), having experienced extensive degradation over the past five centuries [39]. The term Restinga has been used indiscriminately to refer to all types of vegetation that occur in quaternary coastal plains, including the forest vegetation of the lowlands and slopes of the Serra do Mar mountains. Thus, to avoid ambiguity, the term Restinga used in this study is based on topography and follows Rocha and collaborators [40], Souza and collaborators [41], and Franco and collaborators [42] that stated: Restingas are quaternary habitats characterized by sandy soils with high salt concentration covered by predominantly herbaceous and shrubby xerophytic vegetation. Additionally, we also considered in the analysis non-forested sites represented by plains and wet lowlands adjacent to coastal sand ridges, as many amphibian species inhabiting the Restingas commonly use these areas for reproduction and foraging.

**Fig 2:**
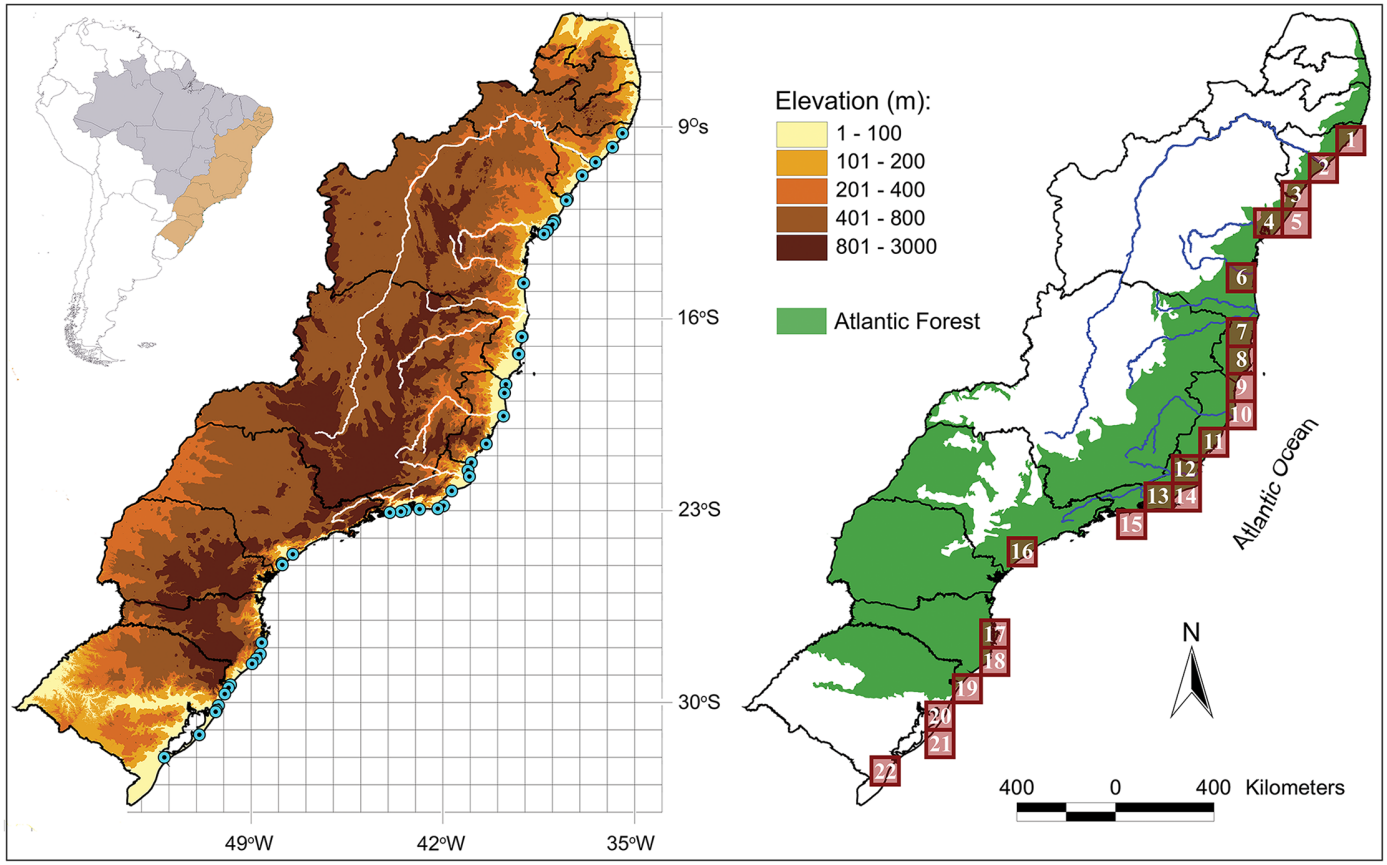
Maps showing the area of study. On left, topographic map showing the point locality sampling of anurans along the Restingas. Right, distribution map of the 22 quadrats considered for the analyses.

The area of Restinga considered here resulted in 22 grids with 1° x 1° resolution (Fig 2), considered the sampling units. We constructed a matrix of presence and absence of anuran species (see Matrix A in S1 Appendix) using the species data contained in each quadrat. This matrix was used in subsequent tests.

### Data sources

Data on anuran composition and their respective geographical distribution were obtained by direct examination of specimens housed in eight Brazilian herpetological collections: Coleção Célio F. B. Haddad, Universidade Estadual Paulista (CFBH); Museu de Ciências e Tecnologia da Pontifícia Universidade Católica do Rio Grande do Sul (MCT-PUCRS); Museu de História Natural Capão da Imbuia, Curitiba, Paraná (MHNCI); Museu Nacional, Rio de Janeiro (MNRJ); Museu de Zoologia da Universidade Estadual de Feira de Santana (MZEFS); Museu de Zoologia da Universidade de São Paulo (MZUSP); and Museu de Zoologia da Universidade Federal da Bahia (UFBA). Additionally, we searched the literature (articles, books, notes on natural history and geographical distribution, environmental impact assessments, management plans, dissertations, and theses) for amphibian species recorded in the studied areas. We critically reviewed information about species and their locations before entering them into the database. In the absence of accurate information on the geographical coordinates for the collection site of any particular specimen, we used Google Earth to obtain approximated geographical coordinates. We considered in the analysis only species that have part of their life cycle associated with the open Restinga. The reliability of the geographic distribution records was verified by consulting the authors and/or collectors of specimens deposited in scientific collections or recorded in the literature. The specimens examined in scientific collections or obtained from literature data are presented in S2 Appendix.

### Analyses

#### Structure of the spatial distribution of anuran species

We searched the main geographical variation patterns in anuran species composition along the eastern Brazilian coast using an indirect gradient analysis. We used the non-metric multidimensional scaling method (NMDS) for reducing the anuran composition dataset (Matrix A) to one or more synthetic axes. Initially, we searched for the best dimensionality to represent the data set. Six dimensions were generated (6D solution) from the Matrix A, by the Bray-Curtis distance coefficient. To avoid the local minima problem [43], we ran 40 starting configurations, using as stability criteria the instability value of 0.000010, 15 iterations to evaluate the stability of the solution and 400 as the maximum number of iterations (see [43]). The Monte Carlo test was used to evaluate whether NMDS extracted a stronger axis than expected by chance. The result indicated that the best solution would be reached by a two dimensional analysis. A new analysis was performed using a two-dimensional (2D) solution with the following settings: 1000 starting configurations, using as stability criteria the instability value of 0,0005, 999 iterations to evaluate the stability of the solution and 500 as the maximum number of iterations. The Monte Carlo test (999 randomizations) was used to evaluate whether NMDS extracted a stronger axis than expected by chance. The NMDS axes were rotated to a new varimax solution [43]. The proportion of variance represented by the NMDS axis, based on the correlation between distance in the ordination space (Euclidian distance) and distance in the original space (Bray-Curtis distance), was obtained by the standardized Mantel test (r). As a last step, a new NMDS analysis was performed with only one dimension (1D solution), using the same 2D configuration settings, to verify whether the 1D solution would be able to synthesize the main pattern of variation in anuran species composition along the eastern Brazilian coast.

We tested the presence of monotonic variation of anuran composition (quadrats; dependent variable) along the latitudinal gradient (independent variable) by single linear regression analysis. The assumption of normal distribution was tested by the Shapiro-Wilk W test and also by the projection of the normal distribution curve over a histogram distribution (observed frequencies); the assumption of linearity was checked by the projection of the variables of interest on a scatter diagram, followed by the ‘runs test’ performed in Prism software version 3.0. The level of significance was set at P ≤ 0.05.

#### Biotic element analyses

The predictions of the vicariance model regarding distribution patterns of amphibians were tested using the biotic element analysis [19,21], which searches for biogeographical units under the vicariance model perspective. Biotic elements (BE) are groups of taxa in which the distributions are significantly more similar to one another than to those of taxa from another group. This analysis was carried out with *prabclus* package in the statistical software R [44] using Matrix A (see S1 Appendix).

Biotic element analysis is based on tests of two predictions of the vicariance model. The first prediction states that division of the ancestral biota should produce groups of taxa that are significantly regionalized [5,19,21]. The second prediction states that closely related species must be found within distinct biotic elements [5,19,21].

Three measures are required to test the null hypothesis that the species distribution ranges are not significantly regionalized (first prediction): a distance measure between the distribution limits of the taxa examined, a statistical test, and a null model to generate sets of random ranges [21]. We chose the geco distance coefficient instead of the Kulczynski distance (default in *prabclus*) because it considers not only the percentage of geographical units shared by taxa, but also the geographical relationships of the occupied units [45–47]. For the required geco coefficient, we used *f* = 0.2. The T test was used based on the assumption that given a significant clustering of scales, the distances between the distribution ranges of the same group will be smaller than those between the ranges of different groups [21]. The distribution of the test statistic under the null model was estimated by a Monte Carlo test.

To test the second prediction of the vicariance model, which states that closely related species must be distributed among distinct biotic elements [19,21], the chi-square test (*X* ^*2*^) was used to analyze the distribution of congeneric species among biotic elements (see [11,28]).

Once it was found that amphibians in the study area are divided into groups of species with significantly regionalized ranges, the next step was to identify the biotic elements. Non-metric multidimensional scaling (NMDS) was applied to the geco distance matrix generated in the previous step. Model-based Gaussian clustering (MBGC) was used on the same geco distance matrix to identify the biotic elements (BE). The percentage of a species distribution in each grid was calculated based on the total species distributed among the different biotic elements. The region with the highest (greater than 75%) percentage of species distribution was defined as the “core area” of each biotic element (see [11,28]).

## Results

A total of 63 anuran species were considered in this study belonging to 18 genera and 6 families (number of species in parenthesis): Bufonidae (8), Craugastoridae (2), Hylidae (36), Leptodactylidae (13), Microhylidae (3), and Odontophrynidae (1). These species were recorded at 40 localities along the eastern Brazilian coast (Fig 2). The list of species included in the analyses is provided in S1 Appendix.

### Structure of the spatial distribution of anuran species

**Fig 3:**
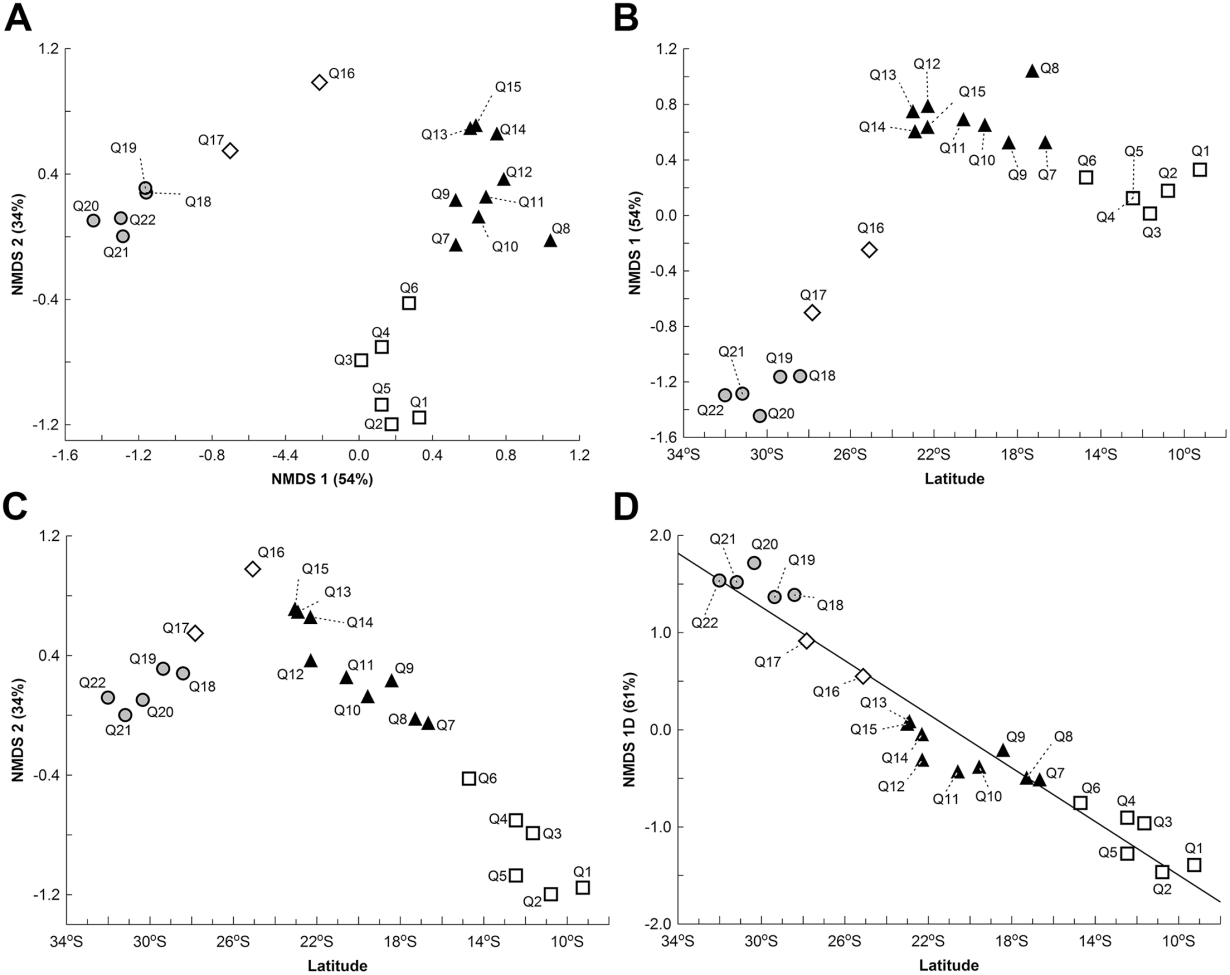
Projection of individual scores resulting from the non-metric multidimensional scaling method (NMDS) for 22 quadrats (sample units). Two-dimensional NMDS solution: (A) NMDS 1 vs. NMDS 2; (B) NMDS 1 vs. latitude; and (C) NMDS 2 vs. latitude. (D) One-dimensional NMDS solution: NMDS vs. latitude; the trend line was obtained from linear regression analysis (F_(1,20)_ = 323.52, R^2^ = 0.94, P < 0.0001). Symbols represent the three main groups resulted from the analysis and that are congruent with biotic elements identified in this study (see Fig 4 for biotic elements): square, northeast biotic element (BE-NE); triangle, southeast biotic element (BE-SE); circle, south biotic element (BE-S); diamond, not included in biotic elements.

The NMDS axes obtained using two dimensions (2D) reflected the structuring in the distribution of the sampling units (SUs) (Fig 3A). The 2D NMDS solution resulted in a stress value of 11.7 and the extracted axes were stronger than expected by chance (Monte Carlo test, P < 0.001). The variance represented by the two NMDS axes explained 88% of the variance present in the original multidimensional space (Mantel test: *r* = 0.94, P < 0.001). This result indicated the presence of three SU groups, representing the northeast, southeast, and south distribution areas. The northeastern and southern SU groups were completely segregated along the first and second axes and the southeastern group was segregated from the southern group in the first axis and from the northeastern group in the second axis. The quadrat Q16 was not clearly related to one of the three recognized groups. Projection of the scores of the NMDS axes onto the latitudinal gradient (Fig 3B and 3C) identified patterns of gradual species turnover (directional clines) along the Atlantic Coast in the latitude ranges 9° S to 25° S (Fig 3C; northeast-southeast gradient) and 22° S to 33° S (Fig 3B, southeast-south gradient). There was convergence of these clines in the latitude range 22° S to 25° S (states of Rio de Janeiro and São Paulo, Fig 3B and 3C).

The one-dimensional (1D) NMDS solution expressed a linear structure in the distribution of anuran species SUs (Fig 3D), but with a high stress value (30.5). Even so, NMDS extracted a stronger axis than expected by chance (Monte Carlo test, P < 0.001). The variance explained by the NMDS axis accounted for 61% of the variance in the original multidimensional space (Mantel test: r = 0.78, P < 0.001). Linear regression analysis carried out between SU ordination (NMDS axis, dependent variable) and the latitudinal gradient (independent variable) indicated a monotonic variation in the SU distribution along the entire length of the coast analyzed (Fig 3D) and a strong dependence of the species distributions (SUs) on the latitudinal gradient (F_(1,20)_ = 323.52, R^2^ = 0.94, P < 0.0001).

### Biotic elements in the Restinga

Biotic element analysis carried out for 63 anuran species corroborated the main vicariance model predictions: (i) the distribution was found to be significantly clustered, forming a regionalized biota along the sandy plains of the beach ridges of the eastern coast of Brazil. The T statistic obtained was 0.146, significantly smaller (P < 0.001) than expected by chance (for 1,000 artificial populations, T ranged from 0.24 to 0.32; mean = 0.28); (ii) species belonging to 15 genera defined the biotic elements: *Aparasphenodon*, *Dendropsophus*, *Dermatonotus*, *Elachistocleis*, *Hypsiboas*, *Leptodactylus*, *Melanophryniscus*, *Phyllodytes*, *Physalaemus*, *Pleurodema*, *Pristimantis*, *Pseudopaludicola*, *Rhinella*, *Scinax*, and *Sphaenorhynchus*. The second prediction of the vicariance model was also corroborated, as closely related species were not significantly clustered in the same biotic element (*X* ^2^ = 42.1053; P = 0.5232).

**Fig 4:**
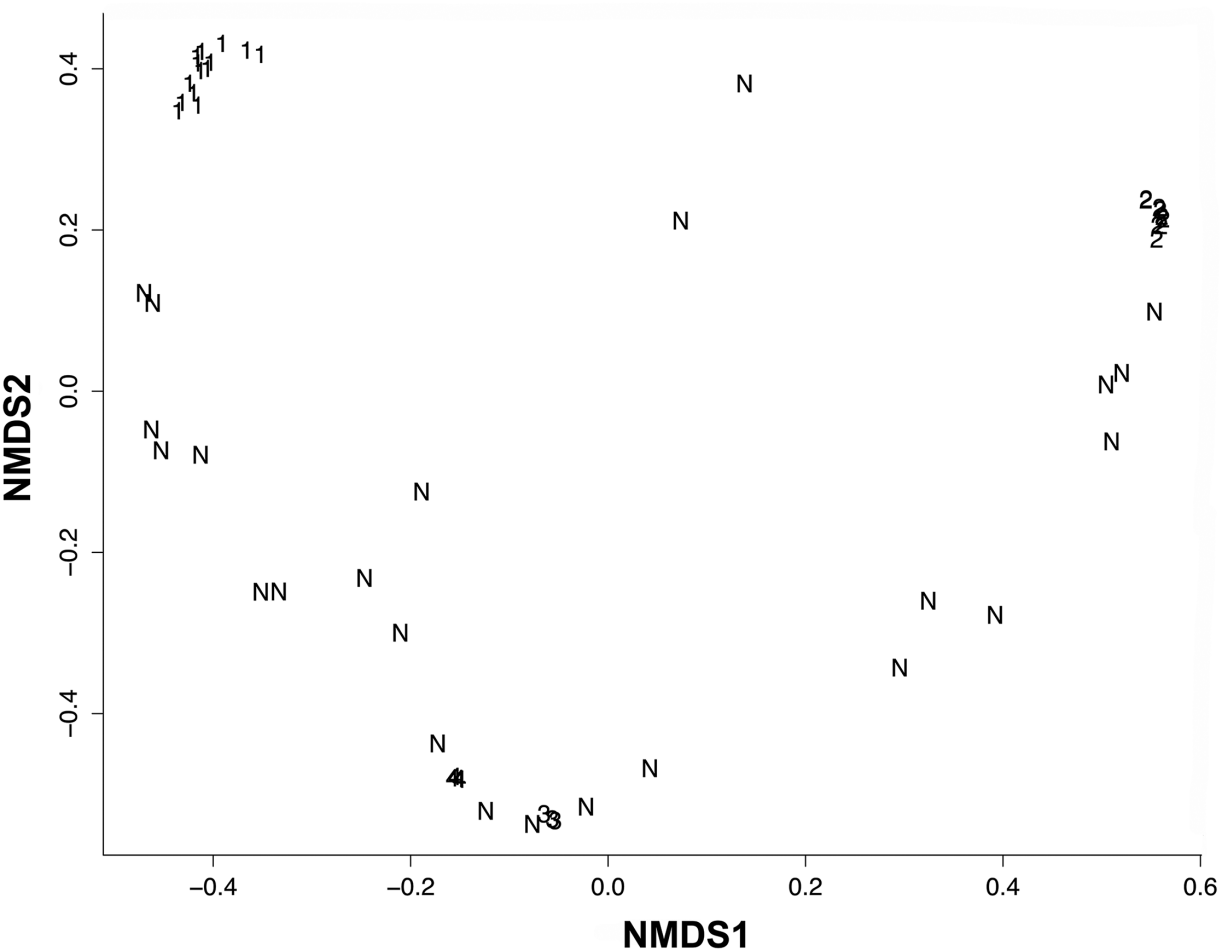
Species clusters in the first two dimensions of nonmetric multidimensional scaling analysis. Data obtained according to the ranges of 63 anuran species from Brazilian Restingas mapped on a 1° x 1° cell grid, analyzed in MCLUST. Characters indicate model-based clustering with noise (N).

**Fig 5:**
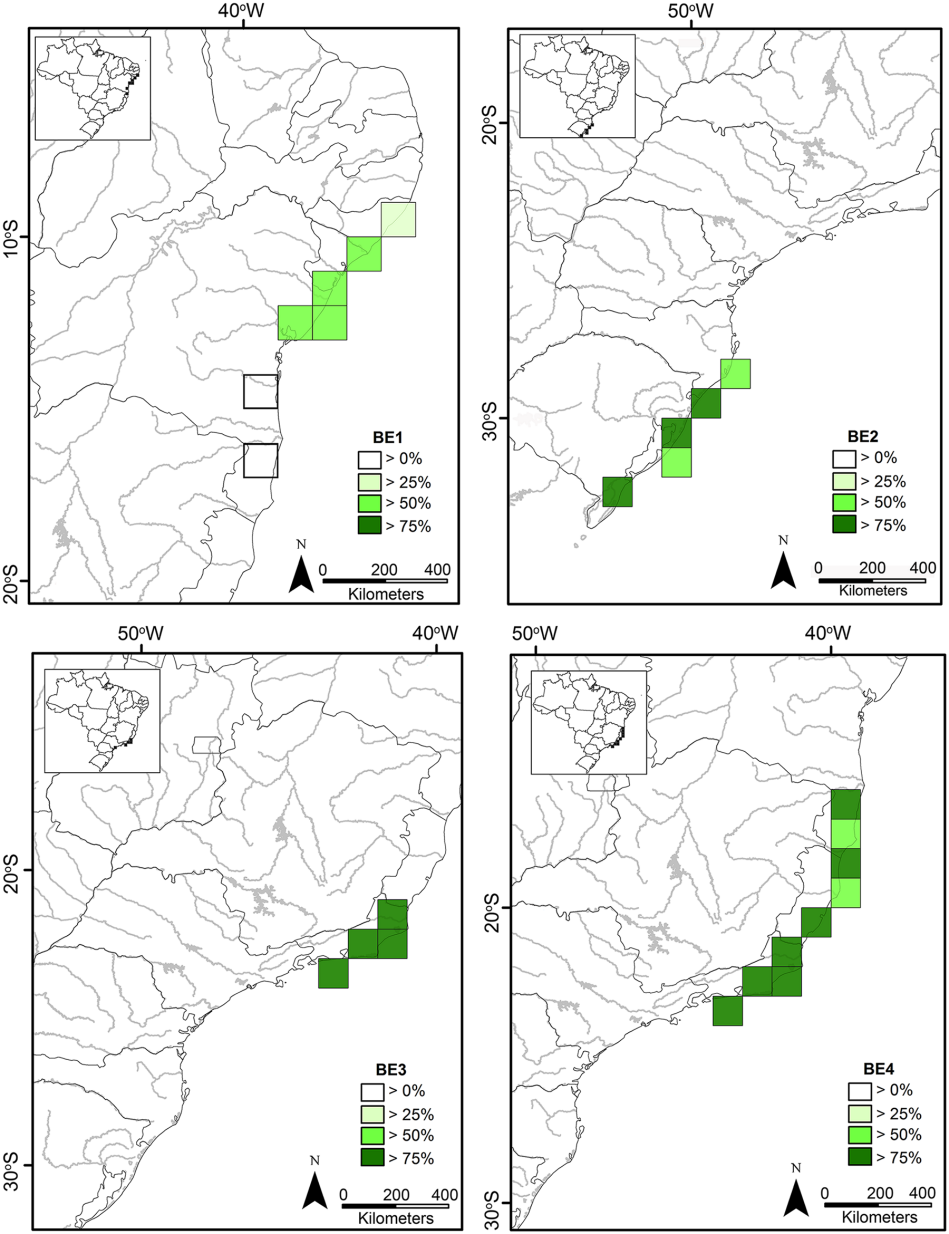
Distribution of the biotic elements (BE) recovered according to the range of 63 anuran species from the Restingas. Each biotic element is comprised by the respective set of quadrats. Shadings indicate the areas where > 75% (= core area), > 50%, > 25% and > 0% of the species of an element are present.

The projection of the individual scores in the reduced space of the two NMDS axes (Fig 4) identified four biotic elements according to the clustering of the species. A total of 25 species (39.7%) were included in the noise component (element “N”, Fig 4), whereas 60.3% of the species (38 species) contributed to the detection of four biotic elements (BEs). The BEs were comprised in three main geographic regions throughout the Brazilian coastline (Fig 5): BE-NE, restricted to the northeastern coast (BE1); BE-SE, in the southeastern coast (BE3 and BE4); BE-S, restricted to the southern coast (BE2). The list of species used in the analysis and their respective occurrences per grid and biotic element are shown in S1 Appendix.

The northeastern biotic element (BE-NE: BE1) extends from the north of the State of Alagoas to the southern coast of the State of Bahia (Fig 5). Fifteen species were identified in the BE-NE region: *Dendropsophus nanus*, *D*. *rubicundulus*, *Dermatonotus muelleri*, *Hypsiboas raniceps*, *Leptodactylus troglodytes*, *L*. *vastus*, *Phyllodytes melanomystax*, *P*. *punctatus*, *Pleurodema diplolister*, *Pristimantis paulodutrai*, *P*. *ramagii*, *Rhinella jimi*, *Scinax auratus*, *S*. *cretatus*, and *S*. *eurydice*. No area in the BE-NE region had more than 75% of richness (core area), but a richer area in the south of the State of Alagoas to the north of the State of Bahia can be recognized.

The southeastern biotic element (BE-SE: BE3 and BE4) extends from the southernmost part of the State of Bahia (below the Jequitinhonha River) to southern Rio de Janeiro State (Fig 5). Five species were identified in the BE3: *Dendropsophus meridianus*, *Leptodactylus mystacinus*, *Rhinella pygmaea*, *Scinax littoreus*, and *S*. *similis*. Five species form the BE4: *Aparasphenodon brunoi*, *Dendropsophus bipunctatus*, *Scinax alter*, *S*. *cuspidatus*, and *Sphaenorhynchus planicola*. The core area of BE3 is coincident with the Restinga of the State of Rio de Janeiro (Q12-Q15). The core areas of BE4 are partially overlapped with the core area of BE3 (Q12-Q15), including also a contiguous area in the southern Espírito Santo State (Q11) and two disjoint areas, one in the southern Bahia State (Q7) and another in the northern Espírito Santo State (Q9). Of all species that occur in the State of Rio de Janeiro, 30% are exclusive to this state, and 70% are shared among Rio de Janeiro, Espírito Santo and Bahia states. The number of anuran species defining BEs in the Restingas of BE-SE increases along the north–south latitudinal gradient from the extreme south of the State of Bahia (Q7; five species, 50%) to the core area in the extreme south of the State of Rio de Janeiro (Q15; nine species, 90%).

The southernmost biotic element (BE-S: BE2) extends along the coasts of the states of Santa Catarina and Rio Grande do Sul (Fig 5). The BE-S consists of 13 species: *Dendropsophus sanborni*, *Elachistocleis bicolor*, *Hypsiboas pulchellus*, *Melanophryniscus dorsalis*, *Physalaemus biligonigerus*, *P*. *gracilis*, *P*. *riograndensis*, *Pseudopaludicola falcipes*, *Rhinella arenarum*, *R*. *dorbignyi*, *R*. *fernandezae*, *Scinax berthae*, and *S*. *squalirostris*. The core area of BE-S covers the south of the State of Santa Catarina to the north of the State of Rio Grande do Sul, as well as an isolated area in the extreme south of the Rio Grande do Sul State. The anuran fauna present in the BE-S predominantly consists of widely distributed species in open areas of wet lowlands in the states of Rio Grande do Sul, Santa Catarina, Paraná, and São Paulo in Brazil, as well as in Uruguay, Argentina and Paraguay. Only the distribution of *Melanophryniscus dorsalis* (states of Santa Catarina and Rio Grande do Sul; [48,49]) is restricted to sandy ridges of the Atlantic Coast.

## Discussion

Our results support the hypothesis that distribution patterns of anuran species along the Restingas of the eastern Brazilian coast are a result of the fragmentation of ancestral biotas as a consequence of vicariant events. The existence of four subgroups (BEs) of amphibian species comprised within three main geographical regions along the Restingas in the eastern Brazilian coast (northeastern, southeastern, and southern biotic elements) confirmed the prediction of the existence of distinct subsets within the study area. The composition of anuran species turned over monotonically along the latitudinal gradient studied. Even considering its monotonic nature, this variation gradient (9°S–33°S) disappears upon reaching 23°S (State of Rio de Janeiro), contributing to the distinction between the southeastern (BE-SE) and southern (BE-S) biotic elements. This break in the variation gradient may be partially explained by the cut-off and discontinuity of the sandy coastal plains in the region between the extreme south of Rio de Janeiro and Santa Catarina states. On the south coast of the State of Rio de Janeiro and north of the State of São Paulo, the anurans are distributed along narrow and fragmented beach ridges (when present), often surrounding small inlets, and most can be group together with the Atlantic Forest anurofauna due to the proximity of the mountainsides that sometimes reach the sea [50]. Moreover, the sandy plains extending along the southern coast of São Paulo to the Municipality of Garopaba (28° S; State of Santa Catarina) are interrupted by the coastal mountains of the Serra do Mar/Paranapiacaba complexes to the north and the mountains in eastern Santa Catarina to the southeast [50]. Thus, these mountain complexes could have been possible barriers to the dispersal of the amphibian species that use the sandy ridges as their main habitat.

Another hypothesis is based on a recent study [36] that could explain the observed break in the north–south variation gradient and the biotic elements identified. According to [36], the following Pleistocene refuges (stable areas) for the Atlantic Forest during the Last Glacial Maximum (approximately 21,000 years ago) were proposed or resurrected: (i) a wide stable area (= Bahia refuge) for the Atlantic Forest in the region between the Doce River to the south and the São Francisco River to the north; (ii) a less stable area to the north of the São Francisco River (= Pernambuco refuge); and (iii) several small stable areas to the south of the Doce River, including two small refuges, one on the coast of the State of Rio de Janeiro near the city of Arraial do Cabo (22.9°S, 42° W) and another on the border between states of Rio de Janeiro and Espírito Santo (ca. 21°S) [36]. Refuges were also proposed within the states of São Paulo and Mato Grosso do Sul. The authors also acknowledged the possible existence of several Atlantic Forest microrefuges in southeastern and southern Brazil (in mountainous areas or valleys) that were recently further supported by Carnaval and collaborators [38].

Carnaval and collaborators [37] subsequently listed, among the model predictions, the absence of genetic patterns of isolation by distance in unstable areas and the presence of these patterns in stable areas (refuges) because, in unstable areas, colonization would have been too recent to restore equilibrium between migration and genetic drift. This prediction was supported by, for example, the genetic patterns observed for *Hypsiboas albomarginatus* and *H*. *faber*. Although we do not have molecular data support, the results obtained here seems to corroborate this prediction because the monotonic latitudinal variation in species composition from northeastern to southeastern Brazil agrees with the expected pattern of isolation by distance, coinciding with the Bahia refuge to a large extent and the patches of coastal refuge predicted for the states of Espírito Santo and, to a lesser extent, Rio de Janeiro. In the south of the coast of São Paulo State, only unstable areas are present according to the model of Carnaval and Moritz [36] and, therefore, the break observed in the monotonic variation coincides with the absence of refuges expected southward from São Paulo and for the southern coast of Brazil.

Additionally, Carnaval and collaborators [38] also provided support for the predictions previously made by Carnaval and Moritz [36] and Carnaval and collaborators [37], and stated that the Atlantic Forest could be divided into two domains or bioclimatic areas (north and south). The eastern Brazilian coast in the northern domain would extend from the State of Rio Grande do Norte to the southern tip of the State of Rio de Janeiro, fully coinciding with the directional cline (monotonic variation) observed for the anuran species of the Restingas considered in this study. The southern domain would extend from the south of the State of Rio Grande do Sul to the south-central coast of the State of Rio de Janeiro (disregarding, therefore, the inland high altitude areas of the state), which clearly coincides with the south–southeast variation of anuran species composition observed in our study. Moreover, both results highlighted the State of Rio de Janeiro as a transition zone for two major axes of biotic composition.

The southern biotic element is located below the 23°S latitude, the break zone in the composition of the anuran species in the study area. This biotic element has (i) a unique faunal composition compared to the southeastern and northeastern biotic elements, (ii) a core area located in southern Brazil, and (iii) a weak but identifiable monotonic variation in anuran species composition from southern Brazil to the south-central coast of São Paulo. These results support the prediction of absence of isolation by distance patterns in unstable areas due to recent colonization, as proposed by Carnaval and collaborators [37], highlighting the disparate composition of the anurofauna of this region compared with adjacent and contiguous areas further north. In addition, the BE-S species composition is more similar to the composition of amphibian communities located in southern and southwestern areas of South America, suggesting that the colonization of this biotic element is associated with the amphibian communities located south and west of the focal coastline, also observed by Garcia and collaborators [50]. This supposed influence on the colonization of this biotic element is also supported by a study of the biodiversity of the regions of Casamento and Butiazais de Tapes lagoons, both located in the coastal plain of Rio Grande do Sul [51]. These authors reported that the coastal plain of this state has a recent geological history and was formed by successive marine transgression and regression events occurring between 400,000 and 5,000 years ago. The flora and fauna of this region would have originated from multiple biogeographical provinces with Atlantic, Pampean, Patagonian, Andean, Chacoan, and Holarctic components [51]. For this reason, the biota of the coastal plain of Rio Grande do Sul would have a low number of endemic species compared to other regions. These assumptions are partially supported by the results of Carnaval and collaborators [38], who reported a low level of phylogeographical endemism for coastal areas located to the south of the State of Paraná and argued that, unlike in the northern area of the Atlantic Forest, endemisms in the southern area are best explained by the current climatic conditions (endemisms in the northern area of the Atlantic Forest would be more related to climate change during the Pleistocene). The current climate acting as the main factor in structuring the southern amphibian fauna was also suggested by Vasconcelos and collaborators [39] that indicated temperature and precipitation seasonality as the main predictors for the amphibians in the Araucaria biogeographical region of the Atlantic Forest, which in turn has a large overlap with the BE-S. In fact, the anuran species that comprise the southern biotic element are present in other morphoclimatic domains in southern South America and only one out of the 13 species of this biotic element is endemic to the southern coast of Brazil, which may indicate a recent colonization pattern.

Most of the core areas of the southeastern biotic elements (BE-SE) are located in a region of possible multiple refuge patches according to the model of Carnaval and Moritz [36], including coastal sandy Restinga areas such as Arraial do Cabo in the State of Rio de Janeiro (23°S) and a small refuge predicted at the boundaries of the states of Espírito Santo and Rio de Janeiro [ca. 21°S, between the Paraíba do Sul (south) and Doce (north) rivers]. The possible existence of this set of refuges located within the states of Rio de Janeiro and Espírito Santo, not contiguous with the Bahia refuge proposed by Carnaval and Moritz [36], could explain the existence of biotic elements distinct from that identified for the northeast of Brazil and equally compatible with formation by vicariant events. The decreasing variation in species number following the south–north latitudinal gradient from the core areas of these elements in southern Rio de Janeiro State to the extreme south of Bahia State, i.e., until reaching the Bahia refuge proposed by Carnaval and Moritz [36], support this statement. Our results and database of distribution suggest the existence of possible secondary contact zones between these biotic elements between the Doce (State of Espírito Santo) and the Jequitinhonha (extreme south of the State of Bahia) rivers. It is also important to note that the Jequitinhonha River area (Q7, Fig 2) is common to two biotic elements (BE-SE and BE-NE), which indicates that this area is a boundary between two biogeographic regions and that this river could be acted as a complex natural barrier.

The model built by Carnaval and Moritz [36], which was later refined [37,38], used both anuran species occurring exclusively in forests (*Brachycephalus* spp., *Chiasmocleis carvalhoi*, *Proceratophrys boiei*, *P*. *renalis*, *Vitreorana eurygnatha*, and *V*. *uranoscopa*) and species inhabiting forest edges and temporary and permanent wetlands in open areas near forested environments (*Dendropsophus elegans*, *Hypsiboas albomarginatus*, *H*. *faber*, *H*. *semilineatus*, *Phyllomedusa burmeisteri* complex, and *Rhinella crucifer* complex). Although not exclusively based on anuran species from lowlands and shrubby restingas along the coast, the inclusion of some species typically found in restingas allowed us to use the model to explain part of the results obtained in the present study. The same statement may be used regarding the biogeographical regions for anurans of the Atlantic Forest proposed by Vansconcelos and collaborators [39]. These biogeographical regions overlap in part with the biotic elements obtained here, but mixing anuran species from forested environments (eg. *Brachycephalus* spp., *Bokermannohyla* spp., *Cycloramphus* spp.) with those from open areas (eg. *Dendropsophus branneri*, *D*. *elegans*, *D*. *minutus*, *Hypsiboas albomarginatus*, *H*. *crepitans*), the biogeographical regions proposed by Vansconcelos and collaborators [39] included regions of the coastal Atlantic Forest without Restinga environments and, therefore, the range extensions of the biogeographical regions (south, southeast, and north) are not the same as those for the biotic elements obtained herein, although they are largely congruent.

The Restinga of northeastern Brazil comprises a biotic element (BE-NE) of distinct nature regarding ecological and biogeographical distribution of its taxa, including species from three distinct morphoclimatic domains (Cerrado, Caatinga, and Tropical Atlantic domains). Eight species (53%) are exclusively from the Tropical Atlantic Domain and inhabit forest borders, lentic water bodies in open areas, and/or terrestrial tank bromeliads in Restingas of northeastern Brazil (*Leptodactylus vastus* [52,53], *Phyllodytes melanomystax* [52,53], *P*. *punctatus* [54,55], *Pristimantis paulodutrai* [52,53], *P*. *ramagii* [49], *Scinax auratus* [56], *S*. *cretatus* [56], and *S*. *eurydice* [52,53]). Seven species (47%) are more common to the Cerrado and Caatinga domains than to the Tropical Atlantic Domain that comprises the coastal areas, and are distributed throughout the northern region of the BE-NE (*Dendropsophus nanus* [57], *D*. *rubicundulus* [58], *Dermatonotus muelleri* [49], *Hypsiboas raniceps* [59], *Leptodactylus troglodytes* [60], *Pleurodema diplolister* [61,62], and *Rhinella jimi* [63]). The overlapping of species typical from the Tropical Atlantic Domain in northeastern Brazil with that from the Caatinga and/or Cerrado domains may be explained by the connection established between the coastal plain (currently belonging to the Tropical Atlantic Domain) and inland Caatinga and Cerrado regions (currently belonging to the Caatinga and Cerrado domains) during the driest periods of the Quaternary (areas therefore under semiarid climate domains) [36,64–65]. The Cerrado remnants in the eastern coast of Brazil are currently small, rare, and sparse, and besides the patches observed in northeastern State of Bahia, other few and small patches occur only in the states of Ceará, Rio Grande do Norte, and Sergipe, always surrounded by other phytophysiognomies or transitional ecological areas [66]. An explanation for the presence of these relictual patches of Cerrado was already identified by Ab’Sáber [65]. He proposed that these Cerrado patches are a result of the expansion of dry climates over the South American landscapes during the last dry period of the Quaternary (12000–18000 years from present). In summary, the core area of the Cerrado Domain [31,66,67], largely distributed over the central part of the continent, was drastically reduced to an enclave in central Brazil, and its northeastern limits became a mosaic of Cerrado and Caatinga patches. Currently, only small patches of the intermixed Cerrado and Caatinga landscapes remain in northeastern Brazil. Ab’Sáber [65] highlighted the small Cerrado patches observed for the center-north region of the State of Bahia, as well as the ones located in the Municipality of Ribeira do Pombal and surroundings, a Cerrado remnant that extends to the south until reaching the Restinga of the Municipality of Camaçari, State of Bahia, in which typical Cerrado species are currently found and that belong to BE-NE (e.g., *Dendropsophus rubicundulus*, *D*. *nanus*, *Hypsiboas raniceps*, and *Dermatonotus muelleri*). The retropicalization subsequent to the Last Glacial Maximum would have been responsible for disjoining the coastal populations from the inland populations throughout the Atlantic Forest strip that again extended along the eastern Brazilian coast.

The identification of biotic elements for the sandy coastal plains of northeastern Brazil is a unique achievement, as the regions so far identified as natural biogeographical units in studies covering the Brazilian northeast have represented, essentially, forested environments (e.g., [68–70]). In the northeastern coast of Brazil, the forests closest to the coast in the states of Pernambuco and Bahia have been systematically identified as areas of endemism for different species groups through multiple analytical methods (e.g., [33,69,70–72]). In studies investigating inland regions where caatinga environments predominate, the regionalization and heterogeneity of the distributions of amphibians [9] and reptiles [11] are essentially associated with (i) mountainous regions that are areas of climatic exception (*sensu* [31,67]) that hold semideciduous high altitude forests and cerrado savanna relicts (e.g., Serra de Maranguape mountains, Chapada Diamantina complex, Ibiapaba–Araripe complex, Serra da Jibóia mountains and Serra do Timbó mountains), (ii) the São Francisco dunes (sand dunes associated with riparian forests) and (iii) the Jequitinhonha valley (riparian forests amid a transition between the Caatinga and Atlantic Forest environments), although Guedes and collaborators [11] identified biotic elements for reptiles coinciding with the boundaries of the lowland Caatinga.

The results obtained in this study are consistent with previous findings by McLennan and Brooks [73] regarding the formation of regionalized biota mosaics. They reported that such mosaics are derived from effects such as vicariance, speciation by peripheral isolation and post-speciation dispersal, and that an area often has histories associated with the species inhabiting it. These historical relationships between the biotic elements identified here and their validity as historical (and not solely geographical) units can be tested in future studies that include the history of the taxa (phylogeny), phylogeography, molecular clocks, and geology in fine-scale biogeographical analyses housing a large number of species (e.g., [11,19,21,74]).

## Supporting Information

S1 Appendix Matrix A. Anuran species (n = 63) per sample quadrat (Q1–Q22) used in the analyses. Sample units were ordered following the Fig 2. Geographic regions of biotic elements are indicated by the following abbreviations: NE—Northeastern, SE—Southeastern, and S—Southern. Biotic elements (BE) are numbered from BE1 to BE4, and N represents model-based clustering with noise. Cells with number one indicate species presence; blank cells, species absence. Anuran families are also indicated: Bu, Bufonidae; Cr, Craugastoridae; Hy, Hylidae; Le, Leptodactylidae; Mi, Microhylidae; Od, Odontophrynidae.
(PDF)

S2 Appendix Anuran species of the Restingas from the eastern Brazilian coast considered as source to our database. Families and species are alphabetically sorted. The data is organized as follows: family, species, country, state, municipality, sample locality, quadrat (Q1–Q22), acronym of scientific collection followed by the respective institutional registration number, and/or bibliographic references (Arabic numbers in brackets).
(PDF)
