# Supplementary material for: Biogeography of Anurans from the Poorly Known and Threatened Coastal Sandplains of Eastern Brazil
Source: PLoS One. 2015 Jun 5;10(6):e0128268. doi: 10.1371/journal.pone.0128268 (PMC4457899; doi:10.1371/journal.pone.0128268)
Supplement: S1 Appendix — Anuran species (n = 63) per sample quadrat (Q1–Q22) used in the analyses. Sample units were ordered following the Fig 2. Geographic regions of biotic elements are indicated by the following abbreviations: NE—Northeastern, SE—Southeastern, and S—Southern. Biotic elements (BE) are numbered from BE1 to BE4, and N represents model-based clustering with noise. Cells with number one indicate species presence; blank cells, species absence. Anuran families are also indicated: Bu, Bufonidae; Cr, Craugastoridae; Hy, Hylidae; Le, Leptodactylidae; Mi, Microhylidae; Od, Odontophrynidae. (PDF) [file pone.0128268.s001.pdf]

S1 Appendix. Matrix A. Anuran species (n = 63) per sample unit/quadrat (Q1–Q22) used in the analyses. Sample units are ordered following NMDS ordination scores and species based on weighted averaging, both from the one dimension solution axis. Geographic regions of biotic elements are indicated by the following abbreviations: NE, Northeastern; SE, Southeastern; and S, Southern. Biotic elements (BE) are numbered from BE1 to BE4, and N represents model-based clustering with noise. Cells with number one indicate species presence; blank cells, species absence. Anuran families are indicated by the following abbreviations: Bu, Bufonidae; Cr, Craugastoridae; Hy, Hylidae; Le, Leptodactylidae; Mi, Microhylidae; Od, Odontophrynidae. (PDF)

Quadrats			Q2	Q1	Q5	Q3	Q4	Q6	Q7	Q8	Q11	Q10	Q12	Q9	Q14	Q15	Q13	Q16	Q17	Q19	Q18	Q21	Q22	Q20
Biotic Elements			BE1	BE1	BE1	BE1	BE1	BE1	BE1/4	BE4	BE4	BE4	BE3/4	BE4	BE3/4	BE3/4	BE3/4	-	-	BE2	BE2	BE2	BE2	BE2
Biotic Element Regions			NE	NE	NE	NE	NE	NE	NE/SE	SE	SE	SE	SE	SE	SE	SE	SE	-	-	S	S	S	S	S
Phyllodytes punctatus	Hy	BE1	1																					
Pristimantis ramagii	Cr	BE1	1	1																				
Rhinella jimi	Bu	BE1	1		1		1																	
Scinax cretatus	Hy	BE1	1	1	1	1	1																	
Dermatonotus muelleri	Mi	BE1		1	1		1																	
Hypsiboas raniceps	Hy	BE1	1		1	1	1																	
Pleurodema diplolister	Le	BE1	1		1	1	1																	
Leptodactylus troglodytes	Le	BE1			1	1																		
Scinax auratus	Hy	BE1	1	1	1	1	1		1															
Leptodactylus vastus	Le	BE1	1		1	1	1		1															
Phyllodytes melanomystax	Hy	BE1			1	1		1																
Leptodactylus natalensis	Le	N		1	1	1	1				1													
Pristimantis paulodutrai	Cr	BE1			1	1	1	1																

Dendropsophus nanus	Hy	BE1				1													
Dendropsophus rubicundulus	Hy	BE1					1												
Scinax melanodactylus	Hy	N	1	1	1	1	1	1	1	1		1							
Scinax eurydice	Hy	BE1					1	1											
Rhinella granulosa	Bu	N	1	1		1	1	1	1		1	1	1	1					
Elachistocleis cesarii	Mi	N					1						1						
Dendropsophus branneri	Hy	N				1	1	1	1		1		1	1					
Phyllodytes luteolus	Hy	N						1	1	1	1	1	1	1					
Sphaenorhynchus palustris	Hy	N							1					1					
Scinax agilis	Hy	N									1	1		1					
Physalaemus marmoratus	Le	N								1			1		1				
Dendropsophus decipiens	Hy	N					1		1		1		1	1	1	1	1		
Aparasphenodon brunoi	Hy	BE4							1	1	1	1	1	1	1	1	1		
Scinax alter	Hy	BE4							1	1	1	1	1	1	1	1	1		
Hypsiboas albomarginatus	Hy	N			1	1	1				1	1	1	1	1	1	1	1	1
Dendropsophus bipunctatus	Hy	BE4							1	1	1		1	1	1	1	1		
Sphaenorhynchus planicola	Hy	BE4							1		1	1	1		1	1	1		
Scinax cuspidatus	Hy	BE4							1		1		1	1	1	1	1		
Dendropsophus elegans	Hy	N						1	1		1	1		1	1	1	1	1	
Phyllomedusa rohdei	Hy	N									1	1			1	1	1		
Leptodactylus mystacinus	Le	BE3											1			1	1		
Dendropsophus meridianus	Hy	BE3											1		1	1	1		
Rhinella pygmaea	Bu	BE3											1		1	1	1		
Scinax similis	Hy	BE3											1		1	1	1		
Scinax littoreus	Hy	BE3													1		1		
Xenohyla truncata	Hy	N													1	1	1		

Leptodactylus marambaiae	Le	N							1								
Dendropsophus minutus	Hy	N	1	1	1	1		1				1	1	1	1	1	
Physalaemus cuvieri	Le	N	1	1								1	1	1			
Rhinella icterica	Bu	N					1			1	1		1				
Scinax tymbamirim	Hy	N							1	1	1	1	1	1			
Scinax imbegue	Hy	N									1	1					
Scinax granulatus	Hy	N										1	1				
Sphaenorhynchus caramaschii	Hy	N										1		1			
Scinax fuscovarius	Hy	N									1		1	1		1	
Scinax berthae	Hy	BE2											1				
Elachistocleis bicolor	Mi	BE2											1	1			
Leptodactylus gracilis	Le	N										1	1	1	1	1	1
Odontophrynus maisuma	Od	N										1	1	1	1	1	1
Melanophryniscus dorsalis	Bu	BE2											1	1		1	
Dendropsophus sanborni	Hy	BE2											1	1	1	1	1
Hypsiboas pulchellus	Hy	BE2											1	1	1	1	1
Physalaemus biligonigerus	Le	BE2											1	1	1	1	1
Physalaemus gracilis	Le	BE2											1	1	1	1	1
Scinax squalirostris	Hy	BE2											1	1	1	1	1
Rhinella arenarum	Bu	BE2											1		1	1	1
Rhinella fernandezae	Bu	BE2											1			1	1
Physalaemus riograndensis	Le	BE2											1				1
Rhinella dorbignyi	Bu	BE2													1	1	1
Pseudopaludicola falcipes	Le	BE2														1	1
