# Supplementary material for: Biogeography of Anurans from the Poorly Known and Threatened Coastal Sandplains of Eastern Brazil
Source: PLoS One. 2015 Jun 5;10(6):e0128268. doi: 10.1371/journal.pone.0128268 (PMC4457899; doi:10.1371/journal.pone.0128268)
Supplement: S2 Appendix — Families and species are alphabetically sorted. The data is organized as follows: family, species, country, state, municipality, sample locality, quadrat (Q1–Q22), acronym of scientific collection followed by the respective institutional registration number, and/or bibliographic references (Arabic numbers in brackets). (PDF) [file pone.0128268.s002.pdf]

S2 Appendix. Anuran species of the Restingas of the eastern Brazilian coast considered as source to our database. Families and species are alphabetically sorted**.** The data is organized as follows: family, species, country, state, municipality, sample locality, quadrat (Q1–Q22), acronym of scientific collection followed by the respective institutional registration number, and/or bibliographic references (Arabic numbers in brackets).

BUFONIDAE: *Melanophryniscus dorsalis*. BRASIL: RIO GRANDE DO SUL: Rio Grande: Ilha dos Marinheiros, Q22, [1]; Torres: Parque Estadual de Itapeva, Q19, MCT-PUC 10350–10353, [2]. SANTA CATARINA: Laguna: Q18, [3]. *Rhinella arenarum*. BRASIL: RIO GRANDE DO SUL: Capão da Canoa, Q19, [4]; Balneário Pinhal: Praia do Magistério, Q20, MCT-PUC 1131, 1212, 1384, 3568, 4242, 4486; Rio Grande: Ilha dos Marinheiros, Q22, [1]; Tavares: Parque Nacional da Lagoa do Peixe, Q21, MCT-PUC 6282; Torres: Q19: MCT-PUC 4967; Parque Estadual de Itapeva, [2]. *Rhinella dorbignyi*. BRASIL: RIO GRANDE DO SUL: Palmares do Sul: Q20, [5]; Rio Grande: Ilha dos Marinheiros, Q22, [1]; Tavares: Parque Nacional da Lagoa do Peixe, Q21, [6]. *Rhinella fernandezae*. BRASIL: RIO GRANDE DO SUL: Balneário Pinhal: Praia do Magistério, Q20, MCT-PUC 5035; Capão da Canoa: Q19, MCT-PUC 7761; Palmares do Sul: Q20, MCT-PUC 1463; Rio Grande: Q22, [5]; Torres: Q19, MCT-PUC 8817. *Rhinella granulosa*. BRASIL: ALAGOAS: Marechal Deodoro: Praia do Saco da Pedra, Q1, MUFAL 8121–8122; Penedo: Várzea da Marituba, Q2, MUFAL 5776; Piaçabuçu: APA Piaçabuçu, Q2, MUFAL 258–261. BAHIA: Camaçari: Restinga de Arembepe, Q4, UFBA 641–663, 997–1001, 9963–9975, 9993, 11862; Conde: Q3, UFBA 12103; Ilhéus: Restinga de Aritaguá, Q6, UFBA 10892; Porto Seguro: Trancoso, Q7, MNRJ 67843. ESPÍRITO SANTO: Conceição da Barra: Parque Estadual de

Itaúnas, Q10, CFBH 2880, [8]; Guarapari: Restinga de Setiba, Q11, [7]; Linhares: Restinga de Regência, Q10, CFBH 26224; Presidente Kennedy: Q12, [9]; São Mateus: Restinga de Guriri, Q9, MNRJ 31271–31272. *Rhinella icterica*. BRASIL: ESPÍRITO SANTO: Conceição da Barra: Parque Estadual de Itaúnas, Q10, [8]. RIO DE JANEIRO: Maricá: Restinga de Maricá, Q13, MNRJ 38989. RIO GRANDE DO SUL: Torres: Parque Estadual de Itapeva, Q19, [2]. SÃO PAULO: Iguape, Q16, [10]. *Rhinella jimi*. BRASIL: ALAGOAS: Penedo: Várzea da Marituba, Q2, MUFAL 5964–5966; Piaçabuçu: APA Piaçabuçu, Q2 MUFAL 222–229. BAHIA: Camaçari: Restinga de Arembepe, Q4, UFBA 567–571, 631–632, 6665–6666, 7817, 9963–9975; Esplanada, Baixio: Q5, UFBA 82; Mata de São João: Praia de Santo Antônio, Vila de Diogo, Q5, UFBA 11208. *Rhinella pygmaea*. BRASIL: ESPÍRITO SANTO: Presidente Kennedy, Q12: [9]; Praia das Neves, [11]. RIO DE JANEIRO: Cabo Frio: Restinga do Peró, Q14, MNRJ 47533–47534; Macaé: Restinga de Jurubatiba, Q14, MNRJ 43816, 66442, [12]; Mangaratiba: Restinga de Marambaia, Q15, MNRJ 20056, [13]; São Francisco de Itabapoana, Q12, MNRJ 45722–45723; São João da Barra: Restinga de Grussaí, Q12, MNRJ 23745–23760, 32446–32453, 55264–55265, [12].

CRAUGASTORIDAE: *Pristimantis paulodutrai*. BRASIL: BAHIA: Camaçari: Restinga de Arembepe, Q4, UFBA 4115; Conde: Q3, UFBA 12094; Jandaíra: Q3, UFBA 11992-11993, 12102; Ilhéus: Restinga de Aritaguá, Q6, UFBA 10814–10824; Mata de São João: Praia do Forte, Q5, UFBA 4833–4839, 5632–5636. *Pristimantis ramagii*. BRASIL: ALAGOAS: Maceió: Q1, MUFAL 7828, 7849, 8026–8031; Penedo: Várzea da Marituba, Q2, MUFAL 5834–5857; Passo de Camaragibe: Q1, MUFAL 7722–7724, 9793.

HYLIDAE: *Aparasphenodon brunoi*. BRASIL: BAHIA: Porto Seguro: Q7, UFBA 6023, 11145–11150, MNRJ 52958, [12]; Prado, Q8: UFBA 9608–9613. ESPÍRITO

SANTO: Conceição da Barra: Parque Estadual de Itaúnas, Q10, CFBH 2394–2396; Guarapari: Restinga de Setiba, Q11, [7,12]; Linhares: Q10, MNRJ 30482–30483; Presidente Kennedy, Q12: [9]; Praia das Neves, [12]. RIO DE JANEIRO: Arraial do Cabo: Restinga de Massambaba, Q13, MNRJ 43400, 45727, [12]; Cabo Frio: Restinga do Peró, Q14, MNRJ 47525; Macaé: Restinga de Jurubatiba, Q14, MNRJ 48670, 66384, [12]; Mangaratiba: Restinga de Marambaia, Q15, MNRJ 20014–20015, 26890, 47552, [13]; Maricá: Restinga de Maricá, Q13, CFBH 17879, MNRJ 57076, 62774, [12]; Rio de Janeiro: Barra da Tijuca, Q15, MNRJ 26893; Restinga de Grumari, Q15, MNRJ 40742, 58063, [12,14]; São Francisco de Itabapoana, Q12, MNRJ 45709; São João da Barra: Restinga de Grussaí, Q12, MNRJ 47542; Iquiparí, Q12, MNRJ 47542. *Dendropsophus bipunctatus*. BRASIL: BAHIA: Porto Seguro, Q7, MNRJ 46484, 47818, 67813, UFBA 11767; Prado: Q8, MNRJ 33123. ESPÍRITO SANTO: Conceição da Barra: Parque Estadual de Itaúnas, Q10, CFBH 4162, [8]; Guarapari: Restinga de Setiba, Q11, [7]; Presidente Kennedy: Praia das Neves, Q12, MNRJ 66454–66456, [12]. RIO DE JANEIRO: Cabo Frio: Restinga do Peró, Q14, MNRJ 47523; Macaé: Restinga de Jurubatiba, Q14, MNRJ 53971; Maricá: Restinga de Maricá, Q13, MNRJ 29240, 29268, 31967, 32022–32033; Mangaratiba: Restinga de Marambaia, Q15, MNRJ 43424–43427; Rio de Janeiro: Barra da Tijuca, Q15, MNRJ 26865–26867, 26868–26870, 26871–26872, 26879. *Dendropsophus branneri*. BRASIL: BAHIA: Conde, Q3, UFBA 7776; Ilhéus: Restinga de Aritaguá, Q6, UFBA 10790; Camaçari: Restinga de Arembepe, Q4, UFBA 579–593, 640, 7534–7553, 8058–8065, 1039–1041, 10989; Entre Rios: Q5, UFBA 5968–5969, 6103; Mata de São João: Praia de Santo Antônio, Vila de Diogo, Q5, UFBA 11207; Porto Seguro: Restinga de Trancoso, Q7, MNRJ 46454, 67831. ESPÍRITO SANTO: Conceição da Barra: Parque Estadual de Itaúnas, Q10, CFBH 4160, [8]; Guarapari: Restinga de Setiba, Q11, [7]; Presidente Kennedy,

Q12: [9]; Praia das Neves, MNRJ 60945–60948. *Dendropsophus decipiens.* BRASIL: BAHIA: Camaçari: Restinga de Arembepe, Q4, UFBA 7614, 1035–1038; Porto Seguro: Q7, UFBA 11769. ESPÍRITO SANTO: Conceição da Barra: Parque Estadual de Itaúnas, Q10, [8]; Guarapari: Restinga de Setiba, Q11, [7]; Presidente Kennedy, Q12: [9]; Praia das Neves, MNRJ 60939–60944, [12]. RIO DE JANEIRO: Arraial do Cabo: Restinga de Massambaba, Q13, MNRJ 45729, [12]; Macaé: Restinga de Jurubatiba, Q14, MNRJ 53918–53919, 66595; Mangaratiba: Restinga de Marambaia, Q15, MNRJ 66595, [12]; Maricá, Q13: Restinga de Itaipuaçu, MNRJ 60694; Restinga de Maricá, MNRJ 29241–29244, 29246, 29260, 31292–31293, 31294, 31998–31999, 32426–32427, 36533; Rio de Janeiro: Barra da Tijuca, Q15, UFBA 531–536, 538–561; Restinga de Grumari, Q15, [14]. *Dendropsophus elegans*. BRASIL: BAHIA: Ilhéus: Restinga de Aritaguá, Q6, UFBA 10688; Porto Seguro, Q7: MNRJ 47806, 67836; Arraial d'Ajuda, UFBA 11179–1179. ESPÍRITO SANTO: Conceição da Barra: Parque Estadual de Itaúnas, Q10, CFBH 2397–2405, [8]; Guarapari: Restinga de Setiba, Q11, [7]. RIO DE JANEIRO: Macaé: Restinga de Jurubatiba, Q14, MNRJ 50466, 53972, 66439; Mangaratiba: Praia do Soco, Q15, MNRJ 43428; Maricá: Restinga de Maricá, Q13, MNRJ 29266, 29300. SÃO PAULO: São Sebastião: Barra do Una, Q16, CFBH 20173. *Dendropsophus meridianus*. BRASIL: RIO DE JANEIRO: Macaé: Restinga de Jurubatiba, Q14, [12]; Maricá: Restinga de Maricá, Q13, MNRJ 29236–29239, 32054–32056, 35927–35929, 36504, 38432–38435; Rio de Janeiro: Barra da Tijuca, Q15, MNRJ 26877–26879; São João da Barra: Restinga de Grussaí, Q12, MNRJ 63496. *Dendropsophus minutus*. BRASIL: BAHIA: Ilhéus: Restinga de Aritaguá, Q6, UFBA 10798; Jandaíra: Costa Azul, Q3, UFBA 11218–11219; Porto Seguro: Arraial d'Ajuda, Q7, UFBA 11768; Entre Rios, Q5, UFBA 5907–5910, 5924–5943, 5963–5966, 6077–6084, 6100–6102, 6119–6130. ESPÍRITO SANTO: Conceição da Barra: Parque

Estadual de Itaúnas, Q10, CFBH 2382–2385, [8]. RIO GRANDE DO SUL: Tavares: Parque Nacional da Lagoa do Peixe, Q21, [6]; Torres: Parque Estadual de Itapeva, Q19, [2]; Rio Grande: Ilha dos Marinheiros, Q22, [1]. SANTA CATARINA: Jaguaruna: Jaboticabeira, Q18, [15]; Palhoça: Baixada do Maciambu, Q17, [16]. *Dendropsophus nanus*. BRASIL: BAHIA: Conde, Q3, UFBA 11836. *Dendropsophus rubicundulus*. BRASIL: BAHIA: Camaçari, Restinga de Arembepe, Q4, UFBA 13634. *Dendropsophus sanborni*. BRASIL: RIO GRANDE DO SUL: Balneário Pinhal: Praia do Magistério, Q20, MCT-PUC 4228–4230; Palmares do Sul: Q20, MCT-PUC 5652; Rio Grande: Ilha dos Marinheiros, Q22, MCT-PUC 3223, [1]; Tavares: Parque Nacional da Lagoa do Peixe, Q21, [6]; Torres: Parque Estadual de Itapeva, Q19, MCT-PUC 8695–8698. SANTA CATARINA: Jaguaruna: Jaboticabeira, Q18, [15]. *Hypsiboas albomarginatus*. BRASIL: BAHIA: Camaçari: Restinga de Arembepe, Q4, UFBA 4750–4754; Entre Rios: Porto Sauipe, Q5, UFBA 5904, 5950–5952, Jandaíra: Costa Azul, Q3, UFBA 11223; Mata de São João, Q5, UFBA 12162. ESPÍRITO SANTO: Conceição da Barra: Parque Estadual de Itaúnas, Q10, [8]; Guarapari: Restinga de Setiba, Q11, [7,12]; Linhares: Restinga de Regência, Q10, MNRJ 29055–29056; Presidente Kennedy, Q12: [9]; Praia das Neves, MNRJ 60329–60331, [12]. RIO DE JANEIRO: Arraial do Cabo: Restinga de Massambaba, Q13, MNRJ 45730; Cabo Frio: Restinga do Peró, Q14, MNRJ 47526; Macaé: Restinga de Jurubatiba, Q14, MNRJ 53967, [12]; Mangaratiba: Restinga de Marambaia, Q15, MNRJ 19951–19952, [13]; Maricá: Restinga de Maricá, Q13, MNRJ 29271–21272; São Francisco de Itabapoana, Q12, MNRJ 45708; São João da Barra: Restinga de Iquiparí, Q12, MNRJ 47549; Rio de Janeiro: Barra da Tijuca, Q15, MNRJ 26868, 26887–26889, 26991–26992; Restinga de Grumari, Q15, [14]. SANTA CATARINA: Palhoça: Baixada do Maciambu, Q17, [16]. *Hypsiboas pulchellus*. BRASIL: RIO GRANDE DO SUL: Balneário Pinhal: Praia do

Magistério, Q20, MCT-PUC 3613–3614; Rio Grande: Estação Ecológica do Taim, Q22, MCT-PUC 3190; Ilha dos Marinheiros, Q22, [1]; Tavares: Parque Nacional da Lagoa do Peixe, Q21, [6]; Torres: Parque Estadual de Itapeva, Q19, MCT-PUC 8458–8459, [2]. SANTA CATARINA: Jaguaruna: Jabuticabeira, Q18, [15]. *Hypsiboas raniceps*. BRASIL: ALAGOAS: Penedo: Várzea da Marituba, Q2, MUFAL 5773–5774. BAHIA: Camaçari: Restinga de Arembepe, Q4, UFBA 7605, 8080, 9646, 9860, 9993; Conde: Beira Mar, Q3, UFBA 11704–11705; Jandaíra: Costa Azul, Q3, UFBA 11213; Entre Rios: Porto Sauipe, Q5, UFBA 5899, 5960; Mata de São João: Vila de Diogo, Praia de Santo Antônio, Q5, UFBA 11198. *Phyllodytes luteolus*. BRASIL: BAHIA: Alcobaça: Q8, [27]; Ilhéus, Q6: UFBA 6531–6532, 7794, [27]; Nova Viçosa: Q8, [27]; Prado, Q8: MNRJ 43099, 47705, [12, 27]; Porto Seguro, Q7: Porto Seguro, [27]; Trancoso, MNRJ 47712, [12]. ESPÍRITO SANTO: Guarapari: Restinga de Setiba, Q11, CFBH 583, 890–896, MNRJ 42413–15, 43096, 43115, 43144, [7, 12]; Linhares: Restinga de Regência, Q10, MNRJ 55391–55404; São Mateus: Restinga de Guriri, Q9, MNRJ 31352, 43085, [12]; Conceição da Barra: Parque Estadual de Itaúnas, Q10, CFBH 4163–4164, [8]. RIO DE JANEIRO: São João da Barra: Restinga de Iquiparí, Q12, MNRJ 47539; Restinga de Grussaí, Q12, MNRJ 42416–42419, [12]. *Phyllodytes melanomystax*. BRASIL: BAHIA: Ilhéus: Restinga de Aritaguá, Q6, UFBA 10854–10855; Mata de São João: Praia do Forte, Q5, UFBA 4911–4915, 4918–4928, 5591–5624, 6648–6657, 10407–10413; Jandaíra: Costa Azul, Q3, UFBA 11217. *Phyllodytes punctatus*. BRASIL: SERGIPE: Areia Branca, Q2, [17]; Santo Amaro das Brotas, Q2, [18]. *Phyllomedusa rohdei*. BRASIL: ESPÍRITO SANTO: Linhares: Q10, 40413–40419, 40858–40868. RIO DE JANEIRO: Arraial do Cabo: Restinga de Massambaba, Q13, MNRJ 45733, 45740; Cabo Frio: Restinga do Peró, Q14, MNRJ 47521, 47535; Maricá: Restinga de Maricá, Q13, MNRJ 502; Rio de Janeiro: Barra da Tijuca, Q15, UFBA 501; Restinga de

Grumari, Q15, [14]. *Scinax agilis*. BRASIL: ESPÍRITO SANTO: Guarapari: Restinga de Setiba, Q11, MNRJ 43094–43095, MZUSP 10245–10454, [7,26]; Linhares: Restinga do Paraju, Q10, CFBH 18052–18056, [26]; São Mateus, Q9, [26]. *Scinax alter*. BRASIL: BAHIA: Porto Seguro, Q7: Arraial d'Ajuda, UFBA 12207; Trancoso, MNRJ 46483, 47815–47817, 67837, [12,22]; Prado: Q8, MNRJ 42420–42436, [22]. ESPÍRITO SANTO: Conceição da Barra, Q10: CFBH 1922–1929, MNRJ 48856–48871, 48873–48897, 48899–488905, 6481–6487; Parque Estadual de Itaúnas, [8]; Guarapari, Q11: Praia de Santa Mônica, MNRJ 33102–33107; Restinga de Setiba, MNRJ 33102–33107, 42013–42014, [7,12]; São Mateus: Restinga de Guriri, Q9, MNRJ 31395–31397, 43084, [12]; Presidente Kennedy, Q12: [9]; Praia das Neves, MNRJ 42015, 60332, 67396, [12]. RIO DE JANEIRO: Arraial do Cabo: Q13, MNRJ 43375–43380, 45728, 45732, 54088, [12]; Rio de Janeiro: Barra da Tijuca, Q15, MNRJ 27041, 27048–27049, 27051; Macaé: Restinga de Jurubatiba, Q14, MNRJ 38972, 66443–66446, 66625–66643, [12]; Mangaratiba: Restinga de Marambaia, Q15, MNRJ 19958, 20059–20060, 20093, [13]; Maricá: Restinga de Maricá, Q13, MNRJ 29254–29258, 29278–29286, 31941–31955, 31956–31966, 32456–32461, 32725–32731, 35304–35322, 36269, 43381–43384, 42437–42485, 43255–43256, [12]; São Francisco de Itabapoana: Q12, MNRJ 45724; São João da Barra, Q12: Restinga de Grussaí, MNRJ 42011–42012, 42184–42185, 55159, [12]; Restinga de Iquiparí, MNRJ 47543. *Scinax auratus*. BRASIL: ALAGOAS: Marechal Deodoro: Praia do Saco, Q1, MUFAL 2765–2768; Penedo: Várzea da Marituba, Q2, MUFAL 2410–2415. BAHIA: Camaçari: Arembepe, Q4, UFBA 1044; Conde: Sítio do Conde, Q3, UFBA 11712; Entre Rios: Porto Sauipe, Q5, UFBA 5970, 6138; Mata de São João: Praia do Forte, Q5, UFBA 4917, 4966–4969, 5844–5845, 5869–5870, 6658; Vila de Diogo, Praia de Santo Antônio, Q5, UFBA 11199; Porto Seguro: Arraial d'Ajuda. Q7, MF Napoli Pers.

observ. *Scinax berthae*. BRASIL: RIO GRANDE DO SUL: Torres: Parque Estadual de Itapeva, Q19, MCT-PUC 8690–8694, [2]. *Scinax cretatus*. BRASIL: ALAGOAS: Maceió: Q1, [21]; Passo de Camaragibe: Q1, [21]. BAHIA: Camaçari: Arembepe, Q4, UFBA 668–676; Lagoa das Dunas, Q4, UFBA 4400–4404; Entre Rios, Q5, UFBA 4385, 5704–5708, 5711–5715, 5722–5746, 6071, 6093, 6104, 6106, 6113–6118; Jandaíra: Costa Azul, Q3, UFBA 11216, 11233; Mata de São João: Praia do Forte, Q5, UFBA 4090, 5882; Praia de Santo Antônio, Vila de Diogo, Q5, UFBA 11200–11201; Salvador: Stella Maris, Q4, UFBA 425–428. SERGIPE: Pirambu: Q2, [21]. *Scinax cuspidatus*. BRASIL: BAHIA: Porto Seguro: Trancoso, Q7, [12]. ESPÍRITO SANTO: Conceição da Barra: Parque Estadual de Itaúnas, Q10, CFBH 2387, [8]; Guarapari: Restinga de Setiba, Q11, MNRJ 42022–42025, [7,12]; Presidente Kennedy: Praia das Neves, Q12, [12]; São Mateus: Restinga de Guriri, Q9, MNRJ 31473, [12]. RIO DE JANEIRO: Arraial do Cabo: Restinga de Massambaba, Q13, MNRJ 45726, [12]; Cabo Frio: Restinga do Peró, Q14, MNRJ 47516–47517; Macaé: Restinga de Jurubatiba, Q14, MNRJ 48671, [12]; Mangaratiba: Restinga de Marambaia, Q15, MNRJ 47553, 47558, 47562; Maricá: Restinga de Maricá, Q13, MNRJ 29050–29052, 29113–29120, 32136–32141, 33229–33248, 35656–33665, 48947–48954, 49041–49043, [12]; Rio de Janeiro: Restinga de Grumari, Q15, [14]; São João da Barra: Restinga de Grussaí, Q12, [12]. *Scinax eurydice*. BRASIL: BAHIA: Ilhéus: Restinga de Aritaguá, Q6, UFBA 10722; Entre Rios: Porto Sauipe, Q5, UFBA 5900, 5903, 6085–6088, 6105. *Scinax fuscovarius*. BRASIL: RIO GRANDE DO SUL: Torres: Parque Estadual de Itapeva, Q19, MCT-PUC 3617, [2]; Rio Grande: Ilha dos Marinheiros, Q22, [1]. SANTA CATARINA: Jaguaruna, Q18, [15]. SÃO PAULO: Cananéia: Q16, CFBH 10682; Iguape: Q16, CFBH 19104; Ilha Comprida: Q16, [10]. *Scinax granulatus*. BRASIL: RIO GRANDE DO SUL: Torres: Parque Estadual de Itapeva, Q19, MCT-PUC 8818.

SANTA CATARINA: Palhoça: Baixada do Maciambu, Q17, [16]. *Scinax imbegue*. BRASIL: SANTA CATARINA: Itajaí: Q17, [22]; Piçarras: Q17, [22]. SÃO PAULO: Bertioga: Q16, [22]; Cananéia: Q16, [22]; Iguape: Q16, [22]. *Scinax littoreus*. BRASIL: RIO DE JANEIRO: Cabo Frio: Restinga do Peró, Q14, MNRJ 47524, 47527–47531; Maricá: Restinga de Maricá, Q13, MNRJ 61117. *Scinax melanodactylus*. BRASIL: ALAGOAS: Barra de Camaragibe, Q1, [19]; Passo de Camaragibe: Q1, [26]. BAHIA: Belmonte, Q7, [26]; Camaçari: Lagoa das Dunas, Q4, UFBA 4394–4399, [26]; Entre Rios: Porto Sauipe, Q5, UFBA 4394–4399, 5911–5915, 5947–5949, [26]; Ilhéus: Q6, UFBA 10420, [Q26]; Mata de São João: Praia do Forte, Q5, UFBA 4106, 4432–4433, 4440, 4929–4939, 4941–4965, 4999, 5662, 5696–5697, 5709–5710, 5853, 5995, 6019, [26]; Praia de Santo Antônio, Vila de Diogo, Q5, UFBA 11203, 11209–112010; Porto Seguro: Porto Seguro, Avenida Beira Mar, Q7, [26], Trancoso, Q7, MNRJ 67838, [12,26]; Prado: Q8, UFBA 9605–9607, [26]. ESPÍRITO SANTO: Conceição da Barra: Parque Estadual de Itaúnas, Q10, CFBH 1938–1944, [26]. SERGIPE: Aracaju: Q3, [26]; Areia Branca, Q2, [20]; Santo Amaro das Brotas, Q2, UFBA 53130, [26]. *Scinax similis*. BRASIL: RIO DE JANEIRO: Rio de Janeiro: Barra da Tijuca, Q15, MNRJ 26918–26926, 26910–26911, 26913–26915, 26927; Macaé: Restinga de Jurubatiba, Q14, MNRJ 38330–38333, 66591–66592; Maricá: Restinga de Maricá, Q13, MNRJ 59910–59913; São João da Barra: Restinga de Grussaí, Q12, MNRJ 35160–35163, 42003–42010, 35160–35163, 47545, 47547–47548, [12]. *Scinax squalirostris*. BRASIL: RIO GRANDE DO SUL: Balneário Pinhal: Praia do Magistério, Q20, MCT-PUC 4226–4227; Rio Grande: Ilha dos Marinheiros, Q22, [1]; Tavares: Parque Nacional da Lagoa do Peixe, Q21, [6]; Torres: Parque Estadual de Itapeva, Q19, MCT-PUC 8688. SANTA CATARINA: Jaguaruna: Q18, [15]. *Scinax tymbamirim*. BRASIL: RIO DE JANEIRO: Rio de Janeiro: Barra da Tijuca, Q15, MNRJ 26900–23907;

Maricá: Q13, [22]. RIO GRANDE DO SUL, Q19, [22]: Arroio do Sal: Arroio Seco; Torres. SANTA CATARINA: Florianópolis, Q17, [22]; Laguna: Q18, pers. comm. M Freire (MCT-PUCRS). SÃO PAULO, Q16, [22]: Bertioga; Cananéia; Iguape. *Sphaenorhynchus caramaschii*. BRASIL: SANTA CATARINA: Jaguaruna, Q18, [15]; Palhoça: Baixada do Maciambu, Q17, [16]. *Sphaenorhynchus palustris*. BRASIL: BAHIA: Porto Seguro: Arraial d'Ajuda, Q7, UFBA 11151–11155, 12200. ESPÍRITO SANTO: Conceição da Barra: Itaúnas, Q10, CFBH 2375–2377. *Sphaenorhynchus planicola*. BRASIL: BAHIA: Porto Seguro: Trancoso, Q7, MNRJ 47811–47812. ESPÍRITO SANTO: Conceição da Barra: Parque Estadual de Itaúnas, Q10, [8]; Guarapari: Restinga de Setiba, Q11, [7]; Linhares: Restinga de Regência, Q10, CFBH 1575–1576; Presidente Kennedy, Q12: [9]; Praia da Neves, MNRJ 66479. RIO DE JANEIRO: Macaé: Restinga de Jurubatiba, Q14, MNRJ 66448; Maricá: Restinga de Itaipuaçu, Q13, MNRJ 60693; Restinga de Maricá, Q13, MNRJ 45314, 58048–58049; Rio de Janeiro: Barra da Tijuca, Q15, MNRJ 26880, 26885. *Xenohyla truncata*. BRASIL: RIO DE JANEIRO: Arraial do Cabo: Restinga de Massambaba, Q13, MNRJ 43396–43398, 45731, [12]; Cabo Frio, Q14: MZUSP 32004–32005; Restinga do Peró, MNRJ 47519; Mangaratiba: Restinga de Marambaia, Q15, [13]; Maricá: Restinga de Maricá, Q13, CFBH 7600–7601, MNRJ 17350–17419, 55856, 59340–59341, 59873–59881, 62773, 42383–42397, UFBA 470–472, [12,23]; Rio das Ostras: Q14, MZUSP 32006–32015); Rio de Janeiro: Barra da Tijuca, Q15, MNRJ 'without registration number'; Restinga de Grumari, Q15, [12].

LEPTODACTYLIDAE: *Leptodactylus gracilis*. BRASIL: RIO GRANDE DO SUL: Arroio do Sal: Arroio Seco, Q19, MCT-PUC 1225; Balneário Pinhal: Praia do Magistério, Q20, MCT-PUC 1071, 1716, 4070, 4857, 4858–4867; Capão da Canoa, Q19, MCT-PUC 4615, 1714–1715; Palmares do Sul, Q20, MCT-PUC 7477; Rio

Grande: Estação Ecológica do Taim, Q22, MCT-PUC 3343; Ilha dos Marinheiros, Q22, [1]; Tavares: Parque Nacional da Lagoa do Peixe, Q21, MCT-PUC 6278, 6280–6281, [6]; Torres: Parque Estadual de Itapeva, Q19, [2]. SANTA CATARINA: Laguna, Q18, pers. comm. M Freire (MCT-PUC); Palhoça: Baixada do Rio Maciambu, Q17, [16]. *Leptodactylus marambaiae*. BRASIL: RIO DE JANEIRO: Mangaratiba: Restinga de Marambaia, Q15, MNRJ 19950, 40743–40746, [13]; São João da Barra: Restinga de Grussaí, Q12, [12]. *Leptodactylus mystacinus*. BRASIL: RIO DE JANEIRO: Arraial do Cabo: Restinga de Massambaba, Q13, MNRJ 45739; Macaé: Restinga de Jurubatiba, Q14, MNRJ 66430; São João da Barra: Restinga de Grussaí, Q12, [12]. *Leptodactylus natalensis*. BRASIL: ALAGOAS: Marechal Deodoro: Praia do Saco, Q1, MUFAL 2480. BAHIA: Camaçari: Restinga de Arembepe, Q4, UFBA 508–530, 639, 1056, 8079; Conde: Q3, UFBA 11212; Entre Rios: Porto Sauipe, Q5, UFBA 5754, 5961–5962; Mata de São João: Praia do Forte, Q5, UFBA 4974–4975, 5847–5852, 5854. ESPÍRITO SANTO: Guarapari: Restinga de Setiba, Q11, [7]. *Leptodactylus troglodytes*. BRASIL: BAHIA: Jandaíra: Costa Azul, Q3, UFBA 11225, 11227–11228; Mata de São João, Q5, UFBA 12159–12160. *Leptodactylus vastus*. BRASIL: ALAGOAS: Piaçabuçu, Q2, MUFAL 309. BAHIA: Camaçari, Q4: Restinga de Arembepe, UFBA 667, 1055; Lagoa das Dunas, UFBA 2381; Jandaíra: Q3, UFBA 12105; Mata de São João: Q5, UFBA 3895, 4081–82, 5026–29, 5871–5874, 5888; Porto Seguro: Arraial d'Ajuda, Q7, MF Napoli pers. observ. *Physalaemus biligonigerus*. BRASIL: SANTA CATARINA: Jaguaruna: Jaboticabeira, Q18, [15]. RIO GRANDE DO SUL: Arroio do Sal: Arroio Seco, Q19, MCT-PUC 2312–2315; Balneário Pinhal: Praia do Magistério, Q20, MCT-PUC 1723–1727; Capão da Canoa, Q19, MCT-PUC 1721–1722; Palmares do Sul, Q20, MCT-PUC 3636; Tavares: Parque Nacional da Lagoa do Peixe, Q21, MCT-PUC 6276, [6]; Torres: Parque Estadual de

Itapeva, Q19, MCT-PUC 8689, [2]; Rio Grande: Ilha dos Marinheiros, Q22, [1]. *Physalaemus cuvieri*. BRASIL: BAHIA: Jandaíra: Costa Azul, Q3, UFBA 11226; Salvador, Q4, UFBA 10579–10582. RIO GRANDE DO SUL: Arroio do Sal: Arroio Seco, Q19, MCT-PUC 3335; Capão da Canoa, Q19, MCT-PUC 1717; Torres: Parque Estadual de Itapeva, Q19, [2]. SANTA CATARINA: Jaguaruna: Jaboticabeira, Q18, [15]; Palhoça: Baixada do Maciambu, Q17, [16]. *Physalaemus gracilis*. BRASIL: RIO GRANDE DO SUL: Arroio do Sal: Arroio Seco, Q19, MCT-PUC 1225; Balneário Pinhal: Praia do Magistério, Q20, MCT-PUC 1716, 1071, 4070, 4857, 4858–4867; Capão da Canoa, Q19, MCT-PUC 1714–1715; Palmares do Sul, Q20, MCT-PUC 7477; Rio Grande: Estação Ecológica do Taim, Q22, MCT-PUC 3343; Ilha dos Marinheiros, Q22, MCT-PUC 6278, 6280–6281, [1]; Tavares: Parque Nacional da Lagoa do Peixe, Q21, [6]; Torres: Parque Estadual de Itapeva, Q19, [2]. *Physalaemus marmoratus*. BRASIL: BAHIA: Prado, Q8, [12]. ESPÍRITO SANTO: Presidente Kennedy: Q12, [9]. RIO DE JANEIRO: Macaé: Restinga de Jurubatiba, Q14, MNRJ 66594; São João da Barra: Restinga de Grussaí, Q12, MNRJ 40880, [12]. *Physalaemus riograndensis*. BRASIL: RIO GRANDE DO SUL: Capão da Canoa, Q19, MCT-PUC 1753; Torres: Parque Estadual de Itapeva, Q19, MCT-PUC 8683–8684, [2]. *Pleurodema diplolister*. BRASIL: BAHIA: Camaçari: Restinga de Arembepe, Q4, UFBA 790–807, 10795, 10987; Entre Rios: Porto Sauipe, Q5, UFBA 5756, 5906, 6068–6070, 6094–6095, 6107, 6137; Jandaíra: Costa Azul, Q3, UFBA 11214, 11229, 11231, 11234–11235; Mata de São João: Praia do Forte, Q5, UFBA 4976–4978, 5625–5627, 5629–5631, 5637; Praia de Santo Antônio, Vila de Diogo, Q5, UFBA 11206; Salvador: Dunas do Abaeté, Q4, MNRJ 61144, UFBA 864–867. SERGIPE: Pirambu, Q2 (pers. comm. EJR Dias, Universidade Federal de Sergipe). *Pseudopaludicola falcipes*. BRASIL: RIO GRANDE

DO SUL: Balneário Pinhal: Praia do Magistério, Q20, MCT-PUC 5653; Palmares do Sul: Q20, MCT-PUC 6062; Rio Grande: Ilha dos Marinheiros, Q22, [1].

MICROHYLIDAE: *Dermatonotus muelleri*. BRASIL: ALAGOAS: Marechal Deodoro: Restinga do Francês, Q1, MUFAL 6438–6440. BAHIA: Camaçari: Restinga de Arembepe, Q4, UFBA 6210, 7856–7859; Mata de São João: Praia do Forte, Q5, UFBA 5628, 7378; Salvador: Dunas do Abaeté, Q4, UFBA 52, 64, 66. *Elachistocleis bicolor*. BRASIL: RIO GRANDE DO SUL: Torres: Q19, MCT-PUC 8685–8687. SANTA CATARINA: Jaguaruna: Jaboticabeira, Q18, [15]. *Elachistocleis cesarii*. BRASIL: BAHIA: Camaçari: Restinga de Arembepe, Q4, UFBA 6233–6235, 6180–6181, 9961. RIO DE JANEIRO: São João da Barra: Atafona, Q12, MNRJ 7978–6981, [25].

ODONTOPHRYNIDAE: *Odontophrynus maisuma*. BRASIL: RIO GRANDE DO SUL: Balneário Pinhal: Praia do Magistério, Q20, MCT-PUC 3617; Tavares: Parque Nacional da Lagoa do Peixe, Q21, MCT-PUC 6285, [6]; Torres: Parque Estadual de Itapeva, Q19, [2]. SANTA CATARINA: Palhoça: Baixada do Maciambu, Q17, [25].

References

1. Quintela FM, Neves LFM, Medvedovisky IG, Santos MB, Oliveira MCLM, Figueiredo MRC (2009) Relação dos anfíbios da Ilha dos Marinheiros, estuário da Lagoa dos Patos, Rio Grande do Sul, BRASIL: Brazilian Journal of Biosciences 7: 231–233. Available: http://www.ufrgs.br/seerbio/ojs/index.php/rbb/article/view/1149. Accessed: 27 October 2014.
2. Colombo P, Kindel A, Vinciprova G, Krause L (2008) Composição e ameaças à conservação dos anfíbios anuros do Parque Estadual de Itapeva, Município de

Torres, Rio Grande do Sul, BRASIL: Biota Neotropica 8: 229–240. Available: http://www.biotaneotropica.org.br/v8n3/en/abstract?inventory+bn01208032008. Accessed: 27 October 2014.

3. Maneyro R, Naya DE, Baldo D (2008) A new species of *Melanophryniscus* (Anura, Bufonidae) from Uruguay. Iheringia, Sér. Zool., Porto Alegre, 98: 189–192.
4. Lema T, Martins LA (2011) Anfíbios do Rio Grande do Sul: catálogo, diagnoses, distribuição, iconografia. Porto Alegre, EDIPUCRS. 196 p.
5. Narvaes P, Rodrigues MT (2009) Taxonomic revision of *Rhinella granulosa* species group (Amphibia, Anura, Bufonidae), with a description of a new species. Arquivos de Zoologia, São Paulo, 40:1–73.
6. Loebmann D, Vieira JP (2005) Relação dos anfíbios do Parque Nacional da Lagoa do Peixe, Rio Grande do Sul, BRASIL: Revista Brasileira de Zoologia 22: 339–341.
7. CEPEMAR - Serviços de Consultoria em Meio Ambiente Ltda., Concessionária Rodovia do Sol (2007) Consolidação dos encartes 1, 2, 3, 4, 5 e 6 do Plano de Manejo da APA de Setiba. Relatório Técnico CPM RT 313/07. Available: http://www.meioambiente.es.gov.br/default.asp?pagina=16708. Accessed: 28 October 2014.
8. CEPEMAR - Serviços de Consultoria em Meio Ambiente Ltda., Petrobrás - Petróleo Brasileiro S.A. (2004) Plano de manejo do Parque Estadual de Itaúnas. Encarte 04 - Caracterização dos fatores ambientais. Meio biótico. Relatório técnico CPM RT 152/02, Revisão 01. Available: http://www.meioambiente.es.gov.br/default.asp?pagina=16707. Accessed: 27 October 2014.

9. Brandt Meio Ambiente, Ferrous Resources do Brasil S.A. (2010) Mineroduto Ferrous: Minas Gerais, Rio de Janeiro e Espírito Santo. Estudo de Impactos Ambientais (Eia). Parte 3: Diagnóstico Ambiental Meio Biótico, 1FRBL006-1-EA-RTE-0004. 248 p.
10. Ramos JZP (2010) Estudo comparativo da taxocenose de anuros de quarto Municípios do Lagamar Paulista. Doctoral thesis, unpublished. Rio Claro, Universidade Estadual Paulista, Programa de Pós-Graduação em Zoologia. 136 p.
11. Marques KIS (2009) Uso do espaço pela lagartixa-de-areia *Liolaemus lutzae* Mertens, 1938 (Liolaemidae), introduzida experimentalmente na Praia das Neves, Espírito Santo. Master Dissertation, unpublished. Seropédica, Universidade Federal Rural do Rio de Janeiro, Programa de Pós-Graduação em Biologia animal. 67 p. Available: http://www.ufrrj.br/posgrad/cpgba/teses/Karina%20Marques,%20Disserta%C3%A7%C3%A3o.pdf. Accessed: 27 October 2014.
12. Rocha CFD, Hatano FH, Vrcibradic D., Van Sluys M (2008) Frog species richness, composition and β-diversity in coastal Brazilian restinga habitats. Brazilian Journal of Biology 68: 101–107.
13. Silva HR, Carvalho ALG, Bittencourt-Silva GB (2008) Frogs of Marambaia: a naturally isolated Restinga and Atlantic Forest remnant of southeastern Brazil. Biota Neotropica 8: 167–174. Available: http://www.biotaneotropica.org.br/v8n4/en/abstract?inventory+bn01808042008. Accessed: 27 October 2014.

14. Telles FBS, Menezes VA, Maia-Carneiro T, Dorigo TA, Winck GR, Rocha CFD (2012) Anurans from the "Restinga" of Parque Natural Municipal de Grumari, state of Rio de Janeiro, southeastern Brazil. Check List 8: 267–1273.
15. Pacheco VS (2012) Taxocenose de anfíbios anuros em biótopos de uma área de Restinga no sul de Santa Catarina. Bachelor's monograph in Biological Sciences, unpublished. Criciúma, Universidade do Extremo Sul Catarinense. 41 p.
16. Wachlevski M, Rocha CFD (2010) Amphibia, Anura, restinga of Baixada do Maciambu, municipality of Palhoça, state of Santa Catarina, southern Brazil. Check List 6: 602–604.
17. Caldas FLS, De-Carvalho CB, Gomes FFA, Freitas EB, Santos RA, Silva BD, et al. (2011) Amphibia, Anura, Hylidae, *Phyllodytes punctatus* Caramaschi and Peixoto, 2004: Distribution extension and first record out of the type locality. Check List 7: 55–56.
18. Caramaschi U, Peixoto OL (2004) A new species of *Phyllodytes* (Anura: Hylidae) from the State of Sergipe, Northeastern Brazil. Amphibia-Reptilia 25: 1–7.
19. Toledo, LF (2005) *Scinax agilis*: geographic distribution. Herpetological Review 36: 77.
20. Passos MA, Bruschi DP, Lima J, Toledo LF (2012) Amphibia, Anura, *Scinax agilis* (Cruz and Peixoto, 1983): Filling gap and new state record. Check List 8: 792–793.
21. Nunes I, Pombal-Jr JP (2011) A new snouted treefrog of the speciose genus *Scinax* Wagler (Anura, Hylidae) from northeastern Brazil. Herpetologica 67: 80–88.

22. Nunes I, Kwet A, Pombal-Jr JP (2012) Taxonomic Revision of the *Scinax alter* Species Complex (Anura: Hylidae). Copeia 2012 (3): 554–569.

23. Silva HR, Carvalho ALG, Bittencourt-Silva GB (2011) Selecting a hiding Place: anuran diversity and the use of bromeliads in a threatened coastal sand dune habitat in Brazil. Biotropica 43: 218–227.

24. Schineider JAP, Teixeira RL (2011) Relacionamento entre anfíbios anuros e bromélias da Restinga de Regência, Linhares, Espírito Santo, BRASIL: Iheringia, Sér. Zool., Porto Alegre, 91: 41–48.

25. Caramaschi, U (2010) Notes on the taxonomic status of *Elachistocleis ovalis* (Schneider, 1799) and description of five new species of *Elachistocleis* Parker, 1927 (Amphibia, Anura, Microhylidae). Boletim do Museu Nacional, Nova Série, Zoologia, Rio de Janeiro 527: 1–30.

26. Lourenço, ACC, Luna MC, Pombal-Jr JP (2014) A new species of the *Scinax catharinae* Group (Anura: Hylidae) from Northeastern Brazil. Zootaxa 3889: 259–276.

27. Salles, ROL, Silva-Soares T (2010) *Phyllodytes luteolus* (Anura, Hylidae) as an alien species in the Rio de Janeiro municipality, State of Rio de Janeiro, Southeastern Brazil. Herpetology Notes 3: 257–258.
